# TRADES: Targeted RNA Demethylation by SunTag System

**DOI:** 10.1002/advs.202001402

**Published:** 2020-08-16

**Authors:** Jing Mo, Zonggui Chen, Shanshan Qin, Shu Li, Chuangang Liu, Lu Zhang, Ruoxi Ran, Ying Kong, Fang Wang, Songmei Liu, Yu Zhou, Xiaolian Zhang, Xiaocheng Weng, Xiang Zhou

**Affiliations:** ^1^ College of Chemistry and Molecular Sciences Wuhan University Wuhan 430072 China; ^2^ The Institute for Advanced Studies College of Life Sciences State Key Laboratory of Virology Wuhan University Wuhan 430072 China; ^3^ State Key Laboratory of Freshwater Ecology and Biotechnology Institute of Hydrobiology Innovation Academy for Seed Design Chinese Academy of Sciences Wuhan 430072 China; ^4^ State Key Laboratory of Virology and Hubei Province Key Laboratory of Allergy and Immunology and Department of Immunology School of Medicine Wuhan University Wuhan 430071 China; ^5^ Department of Clinical Laboratory Center for Gene Diagnosis and Program of Clinical Laboratory Zhongnan Hospital Wuhan University Wuhan 430071 China; ^6^ School of Pharmaceutical Sciences Wuhan University Wuhan 430071 China

**Keywords:** gene expression, N6‐methyladenosine, nucleic acids, RNA modification, site‐specific demethylation

## Abstract

N6‐methyladenosine (m^6^A) is rapidly being studied and uncovered to play a significant role in various biological processes as well as in RNA fate and functions, while the effects of defined m^6^A sites are rarely characterized for the lack of convenient tools. To provide an applicable method to remove m^6^A modification at specific loci, an m^6^A editing system called “targeted RNA demethylation by SunTag system (TRADES)” is engineered. In this system, the targeting element dCas13b is fused to ten copies of GCN4 peptides which can recruit multiple scFv‐fusion RNA demethylase to demethylate the target m^6^A site. Owing to this design, TRADES is more flexible to the indistinct m^6^A sites for its wide editing window. By site‐specific demethylation of messenger RNA (mRNA) SON A2699, the lifetime of SON RNA is successfully prolonged in HeLa cells. Meanwhile, TRADES negligibly influences the lifetime of other non‐targeted transcripts. TRADES also can regulate the gene expression of target transcript in an m^6^A‐dependent manner. Moreover, the interference occuring for HBV and HIV replications demonstrates that the TRADES system holds potential in viral life cycle regulation and clinical applications.

## Graphical abstract

A dCas13b‐SunTag and N6‐methyladenosine (m^6^A) demethylase conjugates system enables the site‐specific demethylation of m^6^ Ain target RNA. It provides convenience to the functional research for its wide editing window and good on‐target capability. Targeted RNA demethylation by the SunTag System successfully regulates the RNA stability, gene expression, and viral life cycles in cells through an m^6^A‐dependent manner.

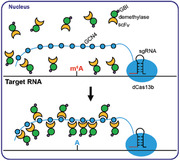

## 1 Introduction

As the most abundant internal (non‐cap) modification in mRNA and lncRNA of all higher eukaryotes, N6‐methyladenosine (m^6^A) is introduced by multicomponent methyltransferase complex containing METTL3, METTL14, WTAP, and so on.^[^ ^1^, ^2^, ^3^ ^]^ In general, m^6^A modification tends to occur at the consensus RRACH motif (R = G or A; H = A, C, or U) and enrich in the vicinity of stop codons of mRNAs.^[^ ^4^, ^5^ ^]^ Owing to the findings of m^6^A demethylases FTO and ALKBH5 which endow m^6^A with dynamic features,^[^ ^6^, ^7^ ^]^ m^6^A is rapidly being studied and uncovered to play a significant role in various biological processes such as stem cell self‐renewal and differentiation,^[^ ^8^ ^]^ control of heat shock response,^[^ ^9^ ^]^ and circadian clock controlling,^[^ ^10^ ^]^ as well as in RNA fate and functions such as mRNA stability,^[^ ^11^, ^12^ ^]^ alternative splicing,^[^ ^13^, ^14^ ^]^ mRNA nuclear export,^[^ ^7^ ^]^ translation,^[^ ^9^, ^15^ ^]^ primary microRNA processing,^[^ ^16^, ^17^ ^]^ and secondary structure switching.^[^ ^18^, ^19^ ^]^ Further investigations also indicate that m^6^A is related to cancer^[^ ^20^ ^]^ and immune system.^[^ ^21^, ^22^, ^23^ ^]^

However, most of current research which relies on globally affecting m^6^A level through adjusting the expression of modifying enzymes could not provide detailed mechanism. Besides, the m^6^A demethylation at specific regions of an individual transcript may have different functions. Point mutation and knock in are two main tools to remove the modification site permanently in site‐specific manner but may bring complicated consequences. So a flexible approach to remove m^6^A at specific site from a transcript of interest is significant to interpret the single RNA methylation event. The clustered regularly interspaced short palindromic repeat (CRISPR) system has been widely used in nucleic acid engineering which showed high potential to address this requirement. The DNA‐targeting Cas protein, the family of Cas9, can be programmed to aid the modification and detection of DNA in their endogenous environment.^[^ ^24^ ^]^ Moreover, an inactivated version of Cas9 (dCas9) nuclease which retains the on‐target ability with sgRNA, but do not break double‐stranded DNA, can be fused with other functional enzymes to facilitate the site‐directed modification of the genome.^[^ ^25^, ^26^ ^]^ The RNA‐targeting Cas proteins, for example, the Cas13 family proteins, have provided a simple and effective way to study and regulate endogenous RNA transcripts. The nuclease LwaCas13a can be programmed to degrade target RNA transcripts efficiently. An in vitro nucleic acid detection platform called “SHERLOCK” also took advantage of the great cleavage activity of Cas13a.^[^ ^27^ ^]^ Meanwhile, catalytically inactive PspCas13b (dCas13b) fused to ADAR2 deaminase domain (ADAR2DD) was used to edit reporter and endogenous transcripts.^[^ ^28^ ^]^

Recently, the fusion of CRISPR‐dCas9 and m^6^A enzymes was developed for programmable m^6^A editing, which opens a new era to elucidate the precise role of RNA modification.^[^ ^29^ ^]^ Rcas9 was also chosen as the RNA‐targeting protein to realize sequence‐specific m^6^A demethylation.^[^ ^30^ ^]^ However, these technologies still need to be improved for two reasons. First, additional PAMer DNA with multiple 2′‐OMe modifications should be co‐transfected via two kinds of transfection reagents that increase the cost and complexity. Second, the distinct m^6^A distribution is still lacking for the imperfect m^6^A sequencing methods, which results many difficulties for sgRNA design in some low‐resolution sites using the fused Cas9‐m^6^A enzyme conjugates with fixed linker. Another approach to manipulate RNA methylation status on specific loci is to engineer a fusion between m^6^A enzymes and the customizable RNA binding protein PUF, which also suffered from the indistinct m^6^A distribution.^[^ ^31^ ^]^

For these limitations and the need to expand the RNA m^6^A editing toolkit, here we developed a method named “targeted RNA demethylation by SunTag system (TRADES)” by using dCas13b, RNA demethylase and SunTag system. SunTag, containing 10 copies of GCN4 peptide which can efficiently recruit scFv‐fusion effector protein, is a signal amplification system. It has been successfully applied in fluorescence imaging, targeted demethylation of specific DNA loci and base editing at endogenous DNA sites.^[^ ^26^, ^32^, ^33^ ^]^ The base editing method named “BE‐PLUS” revealed that SunTag system could increase editing window and enhance fidelity, which encourages us to combine this system with RNA epigenetic editing technology.^[^ ^33^ ^]^ We found that the TRADES system shows wider editing window than direct dCas9‐effector protein conjugates. Thus, the TRADES may be more flexible and potential to target the indistinguishable m^6^A sites. We demonstrated that the TRADES could regulate the stability and expression of target RNA in an m^6^A‐dependent manner. Moreover, the interference occurs for HBV and HIV replications demonstrates that the TRADES system holds the potential in viral life cycle regulation and clinical applications.

## 2 Results

### 2.1 The Generation of TRADES

**Figure 1:**
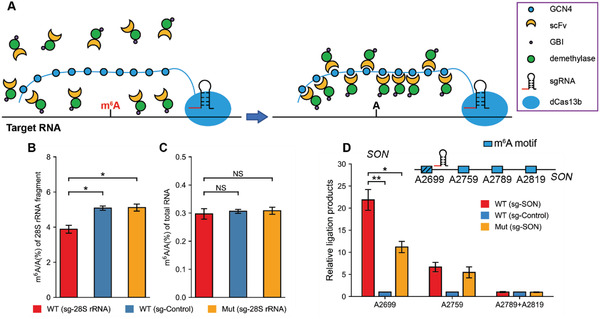
The generation and validation of TRADES system that demethylate RNA at specific loci. A) General overview of targeted RNA demethylation by TRADES system, in which the dCas13b‐GCN4 protein anchored at the target RNA with the guidance of sgRNA. ScFv‐effector proteins are recruited to the binding sites to induce site‐specific demethylation. B) m^6^A/A (%) of 28S rRNA fragment were quantified by LC‐MS/MS. Bars represent mean ± SD from three independent experiments (*n* = 3, *p < 0.05, two‐sided Student's *t*‐test). C) m^6^A/A (%) of total RNA in individual groups were quantified by LC‐MS/MS. In (B) and (C), cells were all transfected with GCN4‐dCas13b. scFv‐XTEN‐FTO (WT), scFv‐XTEN‐FTO^H231A D233A^ (Mut), sgRNA (sg‐28S rRNA), or pc0043 vector (sg‐Control) were transfected as indicated for overall 48 h. Bars represent mean ± SD from three independent experiments (*n* = 3, NS: *p* > 0.05, two‐sided Student's *t*‐test). D) Measurement of m^6^A level on targeted SON A2699, neighboring A2759, A2789, and A2819 in individual groups. Relative ligation products of each group were quantified by qPCR. Cells were all transfected with GCN4‐dCas13b. scFv‐GGS^6^‐FTO (WT), scFv‐GGS^6^‐FTO^H231A D233A^ (Mut), sgRNA (sg‐SON) or pc0043 vector (sg‐Control) were transfected as indicated for overall 48 h. Bars represent mean ± SD from three independent experiments (*n* = 3, *p < 0.05, **p < 0.0001, two‐sided Student's *t*‐test).

We engineered the TRADES system consisting of three parts (**Figure** **1**A). First, a catalytically inactive PspCas13b (dCas13b) is fused to 10 copies of a 19‐amino‐acid GCN4 peptide (Figure S1A, Supporting Information). Then, scFv antibody was fused to the N‐terminal of effector protein, FTO or ALKBH5, which has wild type (WT) and mutant (Mut) versions. Upon fusion to scFv antibody, the effector protein could be recruited to the dCas13b‐GCN4 conjugates. A small soluble tag GB1 was fused to the C‐terminal of effector protein to eliminate protein aggregation (Figure S1B, Supporting Information). The XTEN or (GGS)^6^ short peptide linker was anchored between two function proteins. The linker is critical to adjust the conformation of the recombinant protein to ensure protein activity. Finally, the sgRNA sequence was cloned into pc0043 vector which has U6 promoter. The sgRNA designates the target site through hybridizing to the bases around it and recruiting the dCas13b‐GCN4 conjugates. In each experiment, a panel of sgRNAs with varied spacers was screened toward each different target RNA. Spacer is the region of the sgRNA that hybridized to the target sequence (Figure S1C, Supporting Information). Previous work has shown that m^6^A demethylation caused by FTO is prominent in the cell nucleus. As the proportion of cytoplasmic FTO increases, m^6^A demethylation in HeLa decreases.^[^ ^34^ ^]^ So nuclear localization signal (NLS) sequence was cloned into dCas13b‐GCN4 and scFv‐effector protein‐GBI.

All plasmids were transfected into HeLa cells and their expressions were verified. The western‐blot results showed that these recombinant proteins could be expressed in HeLa cells (Figure S2, Supporting Information). The nuclear localization of these recombinant proteins was investigated using western‐blot from the separated nuclear and cytoplasmic fractions. Because Lamin B is located at nuclear while alpha‐Tubulin mostly located in cytoplasm. Thus, Lamin B and alpha‐Tubulin were selected as references for the separation of nuclear and cytoplasm. As shown in Figure S3, Supporting Information, these recombinant proteins were located in the nuclear mostly.

To investigate whether GCN4‐dCas13b conjugates could recruit scFv‐effector proteins, the co‐localization of GCN4‐dCas13b and scFv‐effector protein was identified by immunofluorescence staining. The results showed that the dCas13b‐GCN4 (red) had a co‐localization with the scFv‐effector proteins (green). DAPI nuclear stain (blue) confirmed the nuclear localization of these recombinant proteins (Figure S4A, Supporting Information). By using the plot profile tool of Image J software and Co‐localization Finder plugin, we have displayed two‐dimensional graphs of the intensities of pixels along a line within the merge image. The *x*‐axis represents distance along the line and the *y*‐axis is the pixel intensity. As shown in Figure S4B, Supporting Information, the GCN4‐dCas13b has a good co‐localization with scFv‐GGS^6^‐ALKBH5. Furthermore, we conducted the co‐immunoprecipitation (Co‐IP) experiments to confirm that GCN4‐dCas13b could specifically capture scFv‐GGS^6^‐effector proteins in transfected cells. Briefly, we transfected HeLa cells with GCN4‐dCas13b and ScFv‐GGS^6^‐FTO or ScFv‐GGS^6^‐ALKBH5 as indicated. The co‐transfection ratio of GCN4‐dCas13b and scFv‐effector protein‐GB1 is 1:1. Two days after transfection, we collected cells to carry out the Co‐IP experiments. As shown in Figure S5A,B, Supporting Information, GCN4‐dCas13b did capture the scFv‐fused demethylases, FTO and ALKBH5. Moreover, the flow through (FT) of the Co‐IP experiment was collected and analyzed by western‐blot. As shown in Figure S5C,D, Supporting Information, the FT from the anti‐His (His‐GCN4‐dCas13b) Co‐IP showed negligible free scFv fused demethylase while the FT from the anti‐IgG (negative control) Co‐IP contained lots of scFv fused demethylase, which re‐confirmed that the scFv‐GGS^6^‐effector proteins were recruited by GCN4‐dCas13b.

### 2.2 The Validation of TRADES System That Demethylates RNA at Specific Loci

We then evaluated the demethylation effect of the TRADES method on the endogenous transcripts. 28S ribosome RNA (rRNA) and mRNA SON transcripts with precise m^6^A sites were chosen to verify the feasibility of TRADES.

It was reported that knockdown of ALKBH5 does not have a statistically significant influence on the m^6^A level of rRNA, which indicated that rRNA is not the major substrate of ALKBH5.^[^ ^7^ ^]^ So FTO was chosen to be the demethylation element for 28S rRNA. The linker of the demethylation part and the spacer length of sgRNA were investigated first. After TRADES treatment, the A4220‐containing fragment in 28S rRNA was isolated from total RNA and digested for LC‐MS/MS analysis followed the published protocol.^[^ ^35^ ^]^ The results showed that scFv‐XTEN‐FTO had a much better demethylation efficiency on 28S rRNA fragment than that of scFv‐GGS^6^‐FTO. The difference may due to the influence of linker on the protein structure and activity. So scFv‐XTEN‐FTO and sg1‐28S rRNA were picked out for further experiments (Figure S6A, Supporting Information). And we compared the demethylation effect of GCN4‐dCas13b and dCas13b‐GCN4 version TRADES to 28S rRNA. As shown in Figure S6B, Supporting Information, GCN4‐dCas13b collaborated better with scFv‐GGS^6^‐FTO than dCas13b‐GCN4. On the other hand, a base‐editing tool called BE‐PLUS which also utilized the Sun‐tag system showed that GCN4‐dCas9 performed more effective than dCas9‐GCN4.^[^ ^33^ ^]^ Based on our initial result and previous report, we chose GCN4‐dCas13b for further experiments in this study. As shown in Figure 1B, the m^6^A level of A4220‐containing fragment decreased by 25% with TRADES system compared to direct FTO over‐expression without sgRNA and mutant FTO control (FTO^H231A D233A^) with sgRNA. While the m^6^A level of total RNA in all treatment groups barely changed (Figure 1C). It is noticed that the demethylation level in 28S rRNA is lower than other RNA species in following researches. We speculated that there may be two reasons. First, the highly structured rRNA may hamper the interaction between sgRNA and target region. Second, as the 28S rRNA is located in the nucleolus, the demethylation effect of TRADES to 28S rRNA may be enhanced by engineered all these proteins in TRADES with nucleolar localization sequences (NoLSs).

For mRNA and lncRNA, which are less abundant compared with rRNA, the m^6^A level should be measured by another reported method named “SELECT.” This method utilized the ability of m^6^A to hinder 1) the single‐base elongation activity of DNA polymerases and 2) the nick ligation efficiency of ligases.^[^ ^36^ ^]^ As a result, the less m^6^A modification would cause higher relative ligation products. Moreover, SELECT could detect the m^6^A level of transcripts with low abundance because it employs qPCR for amplification and quantitation.

Messenger RNA SON A2699 was selected as target site of TRADES. SON has multiple m^6^A modification sites which stand closely. The target A2699 and adjacent A2759, A2789 and A2819 in SON gene were all fit for RRACH motif (Figure 1D). First, we screened the effector proteins and spacer length of sgRNAs to SON A2699. As shown in Figure S6C, Supporting Information, the scFv‐GGS^6^‐FTO (sg4‐SON) group has the best demethylation effect to SON A2699 than other groups. So scFv‐GGS^6^‐FTO and sg4‐SON were selected for further experiments. Then, the m^6^A level of target A2699 and adjacent A2759, A2789 and A2819 were measured by SELECT after TRADES treatment. As shown in Figure 1D, TRADES dramatically demethylated the targeted SON A2699 compared to WT (sg‐Control) and Mut (sg‐SON). FTO‐inactive version of TRADES also decreased the m^6^A level on A2699 and A2759. The demethylation effect of Mut (sg‐SON) on SON A2759 is close to WT (sg‐SON). We speculated that the unanticipated demethylation effect of FTO‐inactive TRADES to SON is caused by endogenous m^6^A demethylase. Upon binding to sgRNA, the secondary structure of SON RNA may change to a status which is more convenient for the access of endogenous m^6^A demethylase. For the SON A2789 and A2819, we detected them in a mixed manner because the sequences of the Up Probe and Down Probe of these two sites are the same in SELECT detection. As shown in Figure 1D, the m^6^A ratio of adjacent SON A2789 and SON A2819 barely changed. Altogether, these results showed that TRADES was effective on SON A2699.

### 2.3 TRADES Features Good On‐Target Capability and Wide Editing Window

m^6^A‐seq and RNA‐seq were performed to evaluate the on‐target ability and demethylation effect of TRADES on SON. To investigate whether the m^6^A peaks we identified contain the m^6^A consensus sequence of RRACH, we analyzed the top 996 significant peaks and found such motif in 882 peaks (Figure S7A, Supporting Information). Moreover, m^6^A peaks were enriched in the vicinity of the stop codon (Figure S7B, Supporting Information). Figure S7C, Supporting Information, showed a significant correlation of gene expression level between two biological replicates. Collectively, these indicated the high quality of our m^6^A‐seq and RNA‐seq data. As shown in Figure S8, Supporting Information, pair‐wise comparison of m^6^A abundance and gene expression level showed few differences which suggests that TRADES has few effects to non‐targeted genes. Moreover, the m^6^A coverage of WT (sg‐SON) at targeted SON A2699 site showed a strong decrease compared to the control which indicates the great demethylation effect of TRADES (Figure S9, Supporting Information). Collectively, TRADES exhibited the capability of site‐specific demethylation and showed low effects toward other untargeted genes.

**Figure 2:**
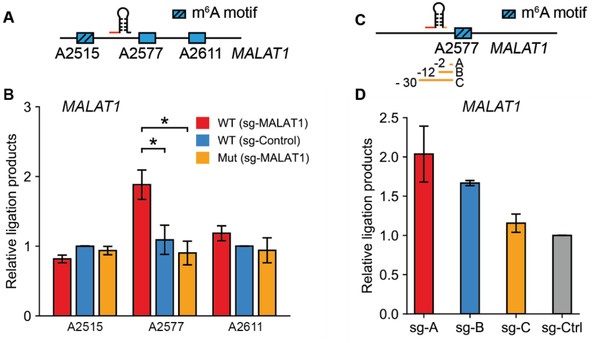
TRADES features wide editing window. A) Schematic representation of the target A2577 site and adjacent A2515 and A2611 sites of MALAT1. B) Measurement of m^6^A level on MALAT1 A2515, A2577, and A2611 in individual groups. Relative ligation products of each group were quantified by qPCR. Cells were all transfected with GCN4‐dCas13b. scFv‐GGS^6^‐ALKBH5 (WT), scFv‐GGS^6^‐ALKBH5^H204A^ (Mut), sgRNA (sg‐MALAT1) or pc0043 vector (sg‐Control) were transfected as indicated for overall 48 h. Bars represent mean ± SD from three independent experiments (*n* = 3, *p < 0.05, two‐sided Student's *t*‐test). C) Schematic representation of the target A2577 site of MALAT1 and a panel of sgRNAs with varied distances to targeting A2577. D) Measurement of m^6^A level on targeted MALAT1 A2577 in individual groups. Relative ligation products of each group were quantified by qPCR. Cells were all transfected with GCN4‐dCas13b and scFv‐GGS^6^‐ALKBH5 (WT). sgRNAs targeting MALAT1 with varied distance to MALAT1 A2577 or pc0043 vector (sg‐Control) was transfected as indicated for overall 48 h. Bars represent mean ± SD from three independent experiments (*n* = 3, two‐sided Student's *t*‐test).

We then utilized SELECT to measure the demethylation effect of TRADES to lncRNA MALAT1 A2577. We screened a panel of sgRNAs targeting MALAT1 A2577 and the effector proteins. The results showed that scFv‐GGS^6^‐ALKBH5 (sg4‐MALAT1) had the best demethylation rate compared with others (Figure S10A, Supporting Information). So sg4‐MALAT1 and scFv‐GGS^6^‐ALKBH5 were selected for further analyses. There are three known m^6^A sites (m^6^A2515, m^6^A2577, and m^6^A2611) on lncRNA MALAT1 transcript from HeLa (**Figure** **2**A). To detect the demethylation effect and on‐target ability of TRADES to MALAT1 A2577, we measured the m^6^A level of these three m^6^A sites in individual treatment. As shown in Figure 2B, the m^6^A level of MALAT1 A2577 was substantially reduced by TRADES compared to direct ALKBH5 overexpression without sgRNA (sg‐Control) and mutant control (ALKBH5^H204A^). While the methylation status of nearby m^6^A sites barely changed after TRADES‐based editing of A2577.

To investigate whether SunTag system could enlarge editing window of TRADES compared to dCas9‐m^6^A enzyme conjugates, we then designed three sgRNAs with 2, 12, 30 nucleotides away from MALAT1 A2577 site, respectively (Figure 2C). We observed efficient demethylation by sgA‐MALAT1 and sgB‐MALAT1, while relative low demethylation efficiency by sgC‐MALAT1 (Figure 2D). In previous dCas9 fusion method, only sgRNA 2 nt away from MALAT1 A2577 has demethylation effect, while the sgRNA 12 nucleotides away from A2577 has no effect, which reveals a narrow editing window.^[^ ^29^ ^]^ So the editing window of TRADES for MALAT1 is much broader than that of previous dCas9 fusion method.^[^ ^29^ ^]^

### 2.4 TRADES Regulates the Target RNA Stability and Expression in an m^6^A‐dependent Manner

m^6^A in regions of the 3′‐UTR could regulate RNA stability through specific recognition by human YTH domain family 2 (YTHDF2) “reader” protein. Upon binding to YTHDF2, mRNA from the translatable pool transferred to mRNA decay sites, such as processing bodies.^[^ ^11^ ^]^ SON, which was known to be m^6^A modified and had been validated for binding by YTHDF2, was selected as the target gene to investigate whether its stability could be influenced after TRADES treatment.

**Figure 3:**
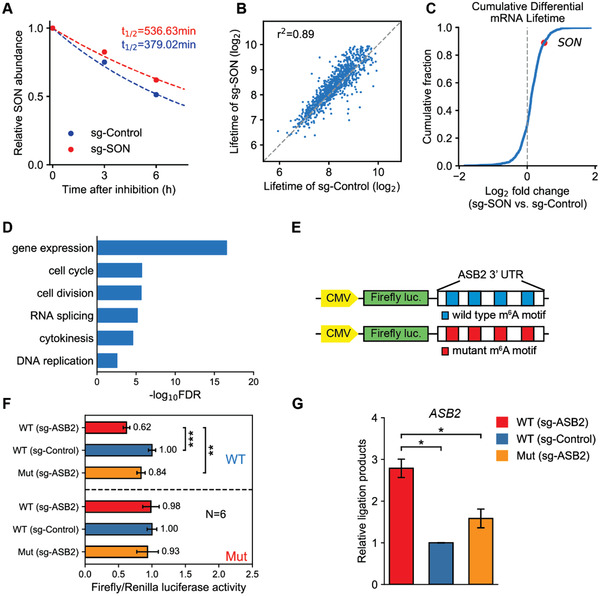
Regulation of target RNA stability and expression in an m^6^A‐dependent manner using TRADES system. A) The dot indicates the relative mRNA content of SON relative to 0 h at 0, 3, and 6 h under WT (sg‐Control) and WT (sg‐SON) processing conditions. The dotted line indicates the fitted degradation curve. B) The dot indicates the pairwise comparison of genes halftime under WT (sg‐Control) and WT (sg‐SON) treatment. C) Cumulative curve indicates the fold‐change (log2) in halftime of all genes under the WT (sg‐Control) and WT (sg‐SON) treatment. In (A)–(C), cells were all transfected with GCN4‐dCas13b and scFv‐GGS^6^‐FTO. sgRNA (sg‐SON) or pc0043 vector (sg‐Control) were transfected as indicated for overall 48 h. D) GO‐enrichment analysis of all the genes which have prolonged lifetime by TRADES treatment. GO term analysis was performed by PANTHER tool, provided by Gene Ontology Consortium. Fisher's exact test was used to adjust the raw *p* value. E) Schematic representation of constructs used in dual‐luciferase analysis. ASB2 3′‐UTR with wild type (blue) or mutant m^6^A (red) motif was cloned at the 3′ downstream of the firefly luciferase. TRADES was applied to targeted demethylate ASB2 3′‐UTR. F) The Firefly/Renilla luciferase activity was accessed by Dual‐Luciferase Reporter Assay System. Cells were all transfected with GCN4‐dCas13b. scFv‐GGS^6^‐FTO (WT), scFv‐GGS^6^‐FTO^H231A D233A^ (Mut), sgRNA (sg‐ASB2), or pc0043 vector (sg‐Control) were transfected as indicated for overall 48 h. Bars represent mean ± SD from three independent sample with two technical replicate experiments (*n* = 6, **p < 0.0001, ***p < 0.000001, two‐sided Student's *t*‐test). G) Measurement of m^6^A level on targeted ASB2 m^6^A site in individual groups. Relative ligation products of each group were quantified by qPCR. Cells were all transfected with GCN4‐dCas13b. scFv‐GGS^6^‐FTO (WT), scFv‐GGS^6^‐FTO^H231A D233A^ (Mut), sgRNA (sg‐ASB2), or pc0043 vector (sg‐Control) were transfected as indicated for overall 48 h. Bars represent mean ± SD from three independent experiments (*n* = 3, two‐sided Student's *t*‐test).

RNA lifetime profiling was conducted by collecting and analyzing RNA‐seq data of targeted SON demethylation and control samples which were treated with actinomycin D at indicated time points. Indeed, TRADES led to prolonged lifetime of its SON mRNA targets in the WT (sg‐SON) group compared with WT (sg‐Control) from RNA‐seq data (**Figure** **3**A), which is corresponding to the previous reported results.^[^ ^9^ ^]^ The lifetime of SON has a larger fold change by TRADES treatment than YTHDF2 knockdown samples.^[^ ^9^ ^]^ Then, the results of RNA lifetime profiling were verified by RT‐qPCR. The transcription of WT (sg‐SON), WT (sg‐control), and Mut (sg‐SON) were inhibited with actinomycin D at the same time points as indicated. Indeed, demethylation of the target RNA prolonged the half‐life of SON transcript than these two controls (WT (sg‐control) and Mut (sg‐SON)), which further confirmed the effect of TRADES system (Figure S11A, Supporting Information). To determine the on‐target ability of TRADES, the pairwise comparison of genes halftime under WT (sg‐Control) and WT (sg‐SON) treatment was conducted. The results revealed that TRADES negligibly influenced the lifetime of other non‐targeted transcripts (Figure 3B). The cumulative curve that indicates the fold‐change (log2) in halftime of all genes under the WT (sg‐Control) and WT (sg‐SON) treatment shows that the lifetime of SON is prolonged to a higher extent than majority of genes (Figure 3C). While some non‐targeted genes did have prolonged lifetime. The involved biological processes of these genes were analyzed by gene ontology (GO) terms. As shown in Figure 3D, most of these genes were involved in gene expression, cell cycle, cell division, RNA splicing, cytokinesis, DNA replication and cellular component organization. As previous work has shown that SON controls the expression of a specific subset of genes which are involved in cell‐cycle progression and genome stability.^[^ ^37^ ^]^ So we speculated that the variation of these genes were related to the regulation of SON. Altogether, these experiments show that TRADES can influence the lifetime of the targeted transcript in an m^6^A‐dependent manner while has little effect to non‐targeted genes.

We next sought to investigate whether TRADES could regulate the gene expression of targeted transcript. FTO has been reported to play an oncogenic role in AML cells. The forced expression of FTO enhances leukemogenesis and inhibits all‐trans‐retinoic acid‐induced leukemia cell differentiation through the epigenetic regulation of its targets, ASB2.^[^ ^38^ ^]^ So, we sought to investigate whether targeted demethylation of ASB2 by TRADES system could influence its expression. Briefly, the dual‐luciferase system which contains firefly luciferase (Fluc) reporter bearing ASB2 3′‐UTR that have intact or mutant m^6^A sites and renilla luciferase reporter as the control were used as targets for demethylation treatment (Figure 3E). First, we screened a panel of sgRNAs to the target ASB2 and selected sg1‐ASB2 for the following experiments (Figure S11B, Supporting Information). As expected, the TRADES system dramatically reduced the Fluc activity of the ASB2 3′‐UTR reporter that has intact m^6^A sites compared with non‐targeting control and mutant control. However, there is nearly no inhibition of Fluc activity in the ASB2 3′‐UTR reporter that bearing mutant m^6^A sites (Figure 3F). Then we calculated the m^6^A abundance of ASB2 targeted site in treatment and control groups by SELECT. As shown in Figure 3G, the levels of m^6^A were reduced by sgRNA targeting ASB2 than that of control. Collectively, these results demonstrated that TRADES system influenced the Fluc activity through demethylating ASB2 3′‐UTR. Overall, the above results from RNA stability assay and dual‐luciferase reporter suggest the TRADES system can target and remove the m^6^A with high efficiency and fidelity.

### 2.5 Effect of Site‐Directed Removal of m^6^A Modification on Virus Replication

Encouraged by the results of programmable demethylation in various kinds of RNA, we intended to investigate the capability of TRADES to viral RNA. HBV is a DNA virus that completes its life cycle through an RNA intermediate called pregenomic RNA (pgRNA). The previous report has shown that there is a distinct m^6^A peak in the region of HBV pgRNA spanning from position 1815–1950. This region contains only one RRACH motif located at position 1905–1909 (GGACA), and A1907 site contains m^6^A methylation. This m^6^A site is encoded in the lower stem of the epsilon stem loop present in all HBV transcripts and is conserved across all known HBV genotypes.^[^ ^23^ ^]^

**Figure 4:**
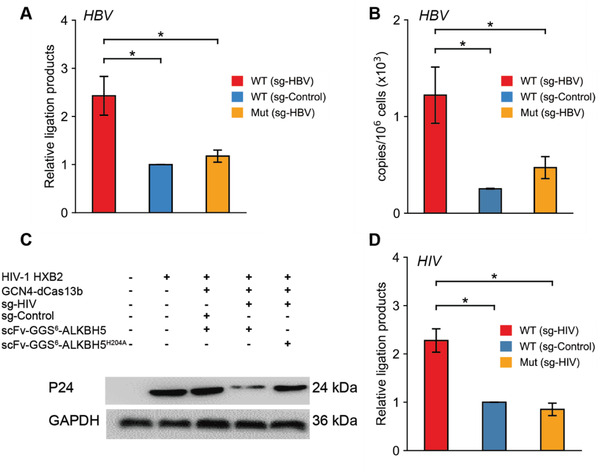
Effect of site‐directed removal of m^6^A modification on Virus replication. A) Measurement of m^6^A level on HBV pgRNA in individual groups. Relative ligation products of each group were quantified by qPCR. Bars represent mean ± SD from three independent experiments (*n* = 3, two‐sided Student's *t*‐test). B) cccDNA copy numbers of HBV in individual groups were measured by micro‐drop digital PCR analysis. In this part, cells were all transfected with GCN4‐dCas13b. scFv‐GGS^6^‐FTO (WT), scFv‐GGS^6^‐FTO^H231A D233A^ (Mut), sgRNA (sg‐HBV), or pc0043 vector (sg‐Control) were transfected as indicated for overall 48 h. Bars represent mean ± SD from three independent experiments (*n* = 3, two‐sided Student's *t*‐test). C) Western‐blot results of HIV‐1 HXB2 P24 intracellular protein expression. Cells were all transfected as indicated for overall 48 h. This experiment was repeated three times with similar results. D) Measurement of m^6^A level on targeted HIV m^6^A site in individual groups. Relative ligation products of each group were quantified by qPCR. Cells were all transfected with GCN4‐dCas13b. scFv‐GGS^6^‐ALKBH5 (WT), scFv‐GGS^6^‐ALKBH5^H204A^(Mut), sgRNA (sg‐HIV), or pc0043 vector (sg‐Control) were transfected as indicated for overall 48 h. Bars represent mean ± SD from three independent experiments (*n* = 3, two‐sided Student's *t*‐test).

To determine whether TRADES could affect HBV replication through epigenetic regulation, we applied TRADES to HBV‐expressing cells. Then we calculated the m^6^A abundance of HBV A1907 in treatment and control groups by SELECT. As shown in **Figure** **4**A, the levels of m^6^A were reduced by sgRNA targeting HBV than that of control. To investigate the influence of TRADES to HBV replication, cellular circular DNA (cccDNA) was isolated and quantified by micro‐drop digital PCR analysis. As shown in Figure 4B, the cccDNA copy numbers of HBV in the wild type FTO group was remarkably increased compared to the mutant FTO^H231A D233A^ transfection groups. Collectively, these results suggest that m^6^A methylation of HBV transcripts negatively regulates HBV cccDNA replication, which is consistent with the conclusions of previous report.^[^ ^23^ ^]^ Thus, we experimentally determined that our method of site‐directed removal of m^6^A modification from HBV pgRNA could modulate HBV replication level.

Intriguingly, previous research demonstrated that ALKBH5 silencing caused a striking increase in viral replication of HIV.^[^ ^21^ ^]^ So we wondered whether TRADES could show potential clinical application to inhibit viral replication by defined m^6^A demethylation. First, we designed a panel of sgRNAs to HIV‐1 HXB2, which has the m^6^A sites in RRE RNAs. Then, HEK293T (CD4+, CCR5) cells were infected with the HIV‐1 HXB2 virus. After 12 h infection, the cells were transfected by plasmids in the TRADES system. Next, viral replication was monitored by western‐blot analysis of p24. As shown in Figure 4C, site‐specifically demethylation of HIV‐1 HXB2 significantly suppressed viral replication compared with cells expressing non‐targeting control sgRNA and mutant ALKBH5 control. Meanwhile, We calculated the targeted‐demethylation effect to HIV by SELECT method. As shown in Figure 4D, the m^6^A level of HIV HBX2 was substantially reduced by TRADES compared to direct ALKBH5 overexpression without sgRNA(WT(sg‐Control)) and mutant control (Mut (sg‐HIV)). Thus, we speculated that the influence of TRADES to HIV replication was due to its site‐specific demethylation effect to HIV HBX2. Altogether, these results suggest that TRADES exhibited high potentiality in some clinical applications especially viral infections.

## 3 Conclusion

As the most abundant RNA modification, m^6^A plays an important role in various biological processes as well as in RNA fate and functions. However, most of current research investigate the function of m^6^A modification through affecting m^6^A level globally and permanently. So we still lack a flexible approach to remove m^6^A with spatial and temporal specificity. Recently, some effective RNA m^6^A editing methods were reported by CRISPR‐dCas9 conjugates and other programmable RNA binding proteins,^[^ ^29^, ^30^, ^31^ ^]^ but with sufficient space for improvement.

In this study, we developed and validated a programmable RNA site‐specific demethylation system–TRADES that relies on the fusion of dCas13b and ten copies of GCN4 peptides which could recruit multiple scFv‐fusion RNA demethylase to demethylate RNA transcript at specific loci. TRADES features wide editing window and good on‐target ability. By site‐specific demethylation of SON, we successfully prolonged the lifetime of SON RNA in HeLa cells. Meanwhile, TRADES negligibly influenced the lifetime of other non‐targeted transcripts. TRADES also could regulate the gene expression of target transcript in an m^6^A‐dependent manner. Moreover, targeted demethylation of HBV pgRNA sufficiently promoted the replication of HBV. Site‐specific demethylation of HIV‐1 HXB2 inhibited the replication of HIV. These studies demonstrate that TRADES system holds the potential in viral life cycle regulation and clinical applications.

Several optimizations remain with the current TRADES system. Such as the size of dCas13b is too large for viral packaging and protein delivery. And continuous delivering of dCas13b which has the bacterial origin may cause immune response.^[^ ^39^ ^]^ We expected that other programmable RNA‐guided RNA target effectors with smaller size and low immunogenicity may overcome these limitations. Moreover, directed evolution of the effector protein perhaps endows TRADES with higher activity.

Generally, TRADES provided a convenient tool to study the functional importance of m^6^A at specific loci in endogenous environment. Because of the excess GCN4 peptides to eliminate the unrecruited effector protein, TRADES may cause less perturbation to other endogenous RNA transcripts than forced expression of effector protein alone. Compared to the dCas9‐demethylase conjugates, TRADES showed wider demethylation window. This difference could be due to the recruitment of multiple copies of demethylase. Based on this, TRADES is more flexible to the indistinct m^6^A sites for its wide editing window, which indeed expanded the RNA m^6^A editing toolkit. Collectively, the efficiency and wide editing window of TRADES suggests that TRADES may be a useful strategy for functional research and clinical application.

## 4 Experimental Section

##### Cloning

The wild type (WT) FTO coding region sequence (CDS) and mutant (Mut) FTO‐CDS (FTO^H231A D233A^) were amplified from pcDNA3‐FTO and pcDNA3‐mutFTO by PCR (Takara, R010Q) and then subsequently cloned into psT1374‐scFv‐APOBEC‐UGI‐GB1 to produce psT1374‐scFv‐XTEN‐FTO (WT or Mut)‐GB1 or psT1374‐scFv‐GGS^6^‐FTO (WT or Mut)‐GB1.

The wild type ALKBH5‐CDS was amplified from pPB‐ALKBH5 by PCR and then subsequently cloned into psT1374‐scFv‐GGS^6^‐GB1 to produce psT1374‐scFv‐GGS^6^‐ALKBH5 (WT)‐GB1 by ClonExpress II One Step Cloning Kit (Vazyme Biotech Co.,Ltd, C112‐01). ALKBH5^H204A^ mutation was introduced by site‐directed Mutagenesis Kit.

For the co‐localization and Co‐immunoprecipitations assay, the His tag in pst1374‐scFv‐gene‐GBI plasmids was replaced by the Cut‐His sequence (Table S1).

The dPspCas13b‐delta 984‐1090 DNA sequence was amplified from pC0050‐CMV‐dPspCas13b‐longlinker‐ADAR2DD (WT) by PCR and then cloned into pFlag‐CMV2 vector to produce pFlag CMV2 dPspCas13b‐delta984‐1090. The GGS^6^ linker Oligo DNA purchased from GeneCreate Co., Ltd. was annealed and then inserted into pFlag CMV2 dPspCas13b‐delta984‐1090 to produce pFlag CMV2 dPspCas13b‐delta984‐1090‐GGS^6^. The GCN4 DNA sequence was amplified from psT1374‐dCas9‐GCN4 by PCR and then cloned into pFlag CMV2 dPspCas13b‐delta984‐1090‐GGS^6^ to produce pFlag CMV2 dPspCas13b‐delta984‐1090‐GGS^6^‐GCN4. The location GCN4 and dPspCas13b‐delta984‐1090 in pFlag CMV2 dPspCas13b‐delta984‐1090‐GGS^6^‐GCN4 was also exchanged to produce pFlag CMV2 GCN4‐GGS^6^‐dPspCas13b‐delta984‐1090. For the co‐localization assay, the Flag tag in pFlag CMV2 dPspCas13b‐delta984‐1090‐GGS^6^‐GCN4 and pFlag CMV2 GCN4‐GGS^6^‐dPspCas13b‐delta984‐1090 was replaced with His tag. For cloning the guide RNAs, the top and bottom oligonucleotides were annealed and inserted into pc0043 vector.

The primers used for subcloning are listed in Table S1, Supporting Information. (F stands for forward primer; R stands for reverse primer; C stands for C‐terminus; N stands for N‐terminus; T stands for top; B stands for bottom).

The dPspCas13b plasmid (pC0050‐CMV‐dPspCas13b‐longlinker‐ADAR2DD(WT)) and sgRNA‐expressing plasmid (pC0043‐PspCas13b crRNA backbone) was a gift from Prof. Feng Zhang (Addgene plasmid no.103866, no.103854). psT1374‐dCas9‐GCN4 (Addgene plasmid no.113026) and psT1374‐scFv‐APOBEC‐UGI‐GB1 (Addgene plasmid no.113029) were gifts from Prof. Xingxu Huang. pcDNA3‐FTO and pcDNA3‐mutFTO were gifts from Prof. Chuan He. pPB‐ALKBH5 was a gift from Prof. Guifang Jia. pFlag‐CMV2 plasmid was a gift from Prof. Xiaolian Zhang. ASB2‐3′‐UTR (wild‐type and mutant) and pRL‐TK were gifts from Prof. Jianjun Chen. pNL4‐3.E‐R‐(CopGFP) and HIV‐env plasmids were gifts from Prof. Binglian Sun. All plasmid sequences were verified by Sanger sequencing.

##### Mammalian Cell Culture and Plasmid Transfection

HEK293T (ATCC) and HeLa (ATCC) cells were maintained using Dulbecco's modified Eagle's medium (DMEM)(high glucose) supplemented with 10% Fetal Bovine Serum (FBS), and 1% penicillin/streptomycin (Beijing Dingguo changsheng Biotechnology Co.,Ltd, GA3502). Plasmids transfection were achieved using lipofectamine 3000 (Invitrogen) following the manufacturer's protocol. The co‐transfection ratio of GCN4‐GGS^6^‐dCas13b, scFv‐effector protein‐GB1 and sgRNA‐expressing plasmid was 1:1:2. HepG2.215 cell line was kindly provided by Prof. Ying Zhu (Wuhan University College of Life Sciences) and maintained in DMEM supplemented with 10% FBS. Rattail collagen (5 µg/100 mL with 120 µL Glacial acetic acid) should be used overnight in cell culture container before cell passage. Each plasmids (pFlagCMV2‐GCN4‐GGS^6^‐dCas13b, SCFV‐GGS^6^‐FTO (WT or Mut) and pc0043‐HBV sgRNA) were transfected into HepG2.215 cells (the transfection ratio was 1:1:2) using NEOFECTTM DNA transfection reagent (Neofect biotech, CHINA) according to the manufacturer's protocol. HEK293T(CD4+,CCR5) cell line was kindly provided by Professor Binglian Sun and maintained in DMEM supplemented with 10% FBS and puromycin (2 µg mL^−1^). Each plasmids (pFlagCMV2‐GCN4‐GGS^6^‐dCas13b, scFv‐GGS^6^‐ALKBH5 (WT or Mutant) and pc0043‐HIV sgRNA) were transfected into HEK293T (CD4+, CCR5) cell line (the transfection ratio was 1:1:2) using NEOFECTTM DNA transfection reagent (Neofect biotech, CHINA) according to the manufacturer's protocol.

##### HIV‐1 HXB2 Virus Production and Infection

HIV‐1 HXB2 virus was produced by transfection of HEK293T cells with the pNL4‐3.E‐R‐(CopGFP) and HIV‐env plasmids (the transfection ratio was 3:1) using Lipofectamine 2000 (Invitrogen), according to the manufacturer's instructions. After 5 h incubation, the medium was replaced with DMEM supplemented with 10% FBS and puromycin (2 µg mL^−1^). Two days after transfection, the virus‐containing supernatant was collected and filtered. The tilter of HIV‐1 HXB2 virus was measured by immunofluorescence. The HEK293T (CD4+,CCR5) cell line was then infected with HIV‐1 HXB2 virus and 12 h later, the cells were centrifuged to remove the virus, washed with PBS and resuspended in DMEM supplemented with 10% FBS and puromycin (2 µg mL^−1^) for further analysis.

##### Western‐Blotting

For western‐blotting, HeLa cells were plated in 12‐well plates and transfected with 1 µg plasmid. After 48 h transfection, cells were washed with PBS and lysed in 50 µL 1 × SDS loading buffer (50 mm Tris‐HCl pH 6.8, 10% glycerol, 2% SDS, 0.1% bromophenol blue, 1% beta‐mercaptoethanol) at room temperature for 10 min. The cell lysate was collected and then boiled at 95 °C for 10 min. The appropriate amount of protein was loaded onto SDS PAGE gels. The separated proteins were transferred onto a PVDF membrane (Millipore) in an ice‐bath for 2 h. Then the PVDF membrane was blocked in 5% (w/v) BSA (Beijing Dingguo changsheng Biotechnology Co.,Ltd, FA016) in TBST (Tris‐buffered saline, 0.1% Tween 20) at room temperature for 1 h. The blot of protein was stained as indicate for at least 12 h at 4 °C. The blot was washed four times with TBST at room temperature for 5 min each, then stained with 1:5000 HRP‐conjugated Affinipure Goat Anti‐Rabbit IgG(H+L) (Proteintech, SA00001‐2) or HRP‐conjugated Affinipure Goat Anti‐Mouse IgG(H+L) (Proteintech, SA00001‐1) in 5% BSA (w/v) in TBST for 1 h at room temperature. The blots were washed four times with TBST at room temperature for 5 min each time and imaged on Molecular Imager ChemiDocTM XRS+ Imaging System (Bio‐Rad) after incubation with Rhea ECL (US Everbright, Inc).

##### RNA Isolation

Total RNA was isolated using TRIzol reagent (Invitrogen, 15596018) following the manufacturer's protocol. mRNA was extracted using Oligo d(T)25 Magnetic Beads (NEB, S1419S) followed by genomic DNA removal using TURBO DNase (Invitrogen, AM2238) treatment. Then RNA was purified by RNA Clean & Concentrator Kits (Zymo Research, R1013). mRNA concentration was measured by NanoDrop.

##### Isolation of a Defined 28S rRNA Fragment

The isolation of a defined 28S rRNA was followed by the published protocols.^[^ ^35^ ^]^ Briefly, to isolate a region of 28S rRNA that contains A4220, biotin labelled oligodeoxynucleotide complementary to C4170–C4210 nucleotide region of 28S rRNA (28S probe: 5′ Biotin‐CTCGCCTTAGGACACCTGCGTTACCGTTTGACAGGTGTAC) was purchased from Takara or obtained by DNA solid‐phase synthesis. To purify synthesized DNA oligo, Crude DNA products were dissolved in DEPC water. The samples were separated on Agilent HPLC system (Thermo Fisher Scientific). Target DNA were evaporated to dryness. The molecular weight was confirmed by LC−ESI mass spectrometry (Sangon Biotech). To obtain RNA: DNA hybrid, 4 µg 28S probe was incubated with about 33 µg of total RNA in 0.3 volumes of hybridization buffer (250 mm HEPES pH 7.0, 500 mm KCl), final 72 µL. The hybridization mixture was boiled at 90 °C for 7 min in water‐bath, slowly cooled down to room temperature overnight. Single‐stranded RNA and DNA were digested with mung bean nuclease (30 units, NEB, #M0250S) and RNaseA (0.5 µg, Thermo Scientific, R1253) over 1 h at 37 °C in 1 × mung bean nuclease buffer (50 mm NaOAc pH 5, 30 mm NaCl, and 1 mm ZnCl_2_). Then wash 30 µL streptavidin C1 beads(Invitrogen, 65002) three times with 500 µL IP buffer (150 mm NaCl, 50 mm Tris, pH 7.9, 0.1% NP40), and combine C1 beads in 100 µL IP buffer with 150 µL RNA‐DNA hybridization, rotate at room temperature for 1 h, and wash 3 times with 500 µL IP buffer, twice with 500 µL H_2_O, then add 30 µL H_2_O, heat at 70 °C, 5 min. It was quickly put on ice for 2 min, and the supernatant was removed to a new tube from magnetic stand; this was the 40 nt RNA fragment.

##### Quantitative PCR

RNA was reverse transcribed to cDNA using the EasyScript One‐Step gDNA Removal and cDNA Synthesis SuperMix (TRANSGEN Biotech, AE311). All qPCR reactions were performed as 20 µL reactions using 2 × Hieff qPCR SYBR Green Master Mix (Yeasen, 11201) and amplified on a CFX‐96 Real‐Time System (Bio‐Rad, USA). Expression levels were obtained by subtraction the housekeeping gene (GAPDH or HPRT1) Ct value from target Ct value and normalizing to the non‐targeting sgRNA (sg‐Control). Relative abundance was determined using 2^−ΔCt^. All assays were performed with at least three biological replicates and three technical replicates. The qPCR primers used in this study are listed in Table S2, Supporting Information. (F stands for forward primer; R stands for reverse primer)

##### Cytoplasm and Nucleus Separation

Cytoplasm and nucleus were separated using Nuclear and Cytoplasmic Extraction Kit (CWBIO, CW0199S) following the manufacturer's protocol. The separation efficiency was validated by western‐blot. The following antibodies were used for western blot analysis: anti‐Flag (rabbit, Sigma), anti‐LaminB1 (ABclonal, A1910), anti‐GAPDH (Proteintech, 10494‐1‐AP ), anti‐Tubulin (ABclonal, AC012).

##### Immunofluorescence Staining and Fluorescence Microscopy

Cells were fixed with 4% paraformaldehyde (Beijing Dingguo changsheng Biotechnology Co.,Ltd, AR‐0211) in PBS at room temperature for 10 min. Cells were then washed with PBS three times and permeabilized with 0.1% TritonX‐100 at room temperature for 10 min. Cells were washed again three times with PBS and blocked for 3 h with 3% (w/v) BSA in TBST at room temperature. Cells were then incubated with primary antibodies (Rabbit Flag antibody, Sigma‐Aldrich, F7425, 20 µg; Mouse anti‐His antibody, ABclonal, AE003 1:200 dilution) in 3% (w/v) BSA for 1 h at room temperature. After washing five times with PBS, cells were incubated with secondary antibodies (Alexa Fluor 488 anti‐rabbit IgG, Cell Signaling Technology, 4412S, 1:2 000 dilution; Alexa Fluor 594 anti‐mouse IgG, Invitrogen, A‐11004, 1:1 000 dilution) in 5% (w/v) BSA for 1 h. After washing five times with PBS, cells were incubated with 1 µg mL^−1^ DAPI (Solarbio, C0060) for 15 min. Cells were then washed five times and imaged.

The cells were imaged immediately by confocal laser scanning microscope (CLSM, PerkinElmer UltraVIEW VoX, Germany). For DAPI imaging, the excitation was 405 nm, and the emission filter was 447 nm (w60); for Alexa Fluor 488 imaging, the excitation was 488 nm, and the emission filter was 525 nm (w50). for Alexa Fluor 561 imaging, the excitation was 561 nm, and the emission filter was 600 nm (w52).

##### LC‐MS/MS

50 ng of mRNA, 28S rRNA fragment or total RNA was digested by nuclease P1 (1 U, Sigma‐Aldrich) in 25 µL of buffer containing 10 mm NaCl and 2 mm of ZnCl_2_ at 37 °C for 2 h, which was followed by addition of NH_4_HCO_3_ (1 M, 2.5 µL, freshly made) and Shrimp alkaline phosphatase (1 U, NEB, M0371S) and additional incubation at 37 °C for 2 h. Samples were centrifuged at 12 000 × *g* at room temperature for 20 min; 3 µL of solution was loaded into Shimadzu LC‐MS/MS system (LCMS‐8050, SHIMADZU). Nucleosides were quantified by using retention time and nucleoside to base ion mass transitions of 282.1 to 150.1 (m^6^A), and 268 to 136 (A).

##### Quantification of m^6^A Level at Single Base Using SELECT

The procedure was adapted from the previous report.^[^ ^36^ ^]^ First, 1 µg total RNA were mixed with 40 nm Up Primer, 40 nm Down Primer and 5 µm dTTP (Takara, 4029) in 17 µL 1 × CutSmart buffer (50 mm KAc, 20 mm Tris‐HAc, 10 mm MgAc_2_, 100 µg mL^−1^ BSA, pH 7.9 @ 25 °C). The RNA and primers were annealed by incubating mixture at a temperature gradient: 90 °C for 1 min, 80 °C for 1 min, 70 °C for 1 min, 60 °C for 1 min, 50 °C for 1 min, and then 40 °C for 6 min. Subsequently, a 3 µL of mixture containing 0.01 U Bst 2.0 DNA polymerase (NEB, M0537s), 0.5 U SplintR ligase (NEB, M0375s) and 10 nmol ATP (Solarbio, IA0590) were added in the former mixture to the final volume 20 µL. The final reaction mixture was incubated at 40 °C for 20 min, denatured at 80 °C for 20 min and kept at 4 °C. Afterward, quantitative real‐time PCR (qPCR) reaction was amplified on a CFX‐96 Real‐Time System (Bio‐Rad, USA) . The 20 µL qPCR reaction was composed of 2 × Hieff qPCR SYBR Green Master Mix (Yeasen), 200 nm qPCRF primer, 200 nm qPCRR primer, 2 µL of the final reaction mixture and ddH_2_O. qPCR was run at the following condition: 95 °C, 5 min; (95 °C, 10 s; 60 °C, 35 s) × 40 cycles; 95 °C, 15 s; 60 °C, 1 min; 95 °C, 15 s (collect fluorescence at a ramping rate of 0.05 °C s^−1^; 4 °C, hold. Data was analyzed by QuantStudioTM Real‐Time PCR Software v1.3. The primers used in SELECT method are listed in Table S3, Supporting Information. (F stands for forward primer; R stands for reverse primer; Uppercase represents common adapter sequence for qPCR; Lowercase represents the sequence that was complementary to detected RNA)

##### m^6^A Immunoprecipitation

Total RNA was isolated by TRIzol reagent (Invitrogen). PolyA‐RNA was isolated by two rounds of ployA selection with Oligo d(T)25 Magnetic Beads (NEB, S1419S) from total RNA and followed by additional TURBO DNase treatment. mRNA was purified by RNA Clean & Concentrator Kits and then fragmented to about 100 nt using RNA Fragmentation Reagents (Invitrogen, AM8740). The fragmented mRNA was purified by using RNA Clean & Concentrator Kits. While 100 ng mRNA was saved as input sample, the rest mRNA was used for m^6^A‐immunoprecipitation. Briefly, nearly 5 µg mRNA was incubated with m^6^A antibody (Synaptic Systems, 202 003) and diluted into 500 µL IP buffer (150 mm NaCl, 0.1% Igepal CA‐630, 10 mm Tris, pH 7.4, 100 U RNase inhibitor). The mixture was rotated at 4 °C for 2 h, following Dynabeads Protein A (Invitrogen, 10002D) was added into the solution and rotated for another 2 h at 4 °C. After four times washing by IP buffer, the m^6^A IP portion was eluted twice by 100 µL m^6^A‐elute buffer (IP buffer, 6.7 mm m^6^A, 30 U RNase inhibitor) with incubating and shaking at 4 °C for 1 h. Finally, m^6^A IP mRNA was recovered by RNA Clean & Concentrator Kits and the RNA concentration was measured with Qubit RNA HS Assay Kit (Invitrogen, Q32855). Sequencing libraries of input and IP samples were prepared using NEBNext Ultra II RNA Library Prep Kit. mRNA m^6^A profiling was generated from two biological replicates.

##### RNA Stability Assay and Sequencing for mRNA Lifetime

HeLa cells were seeded into 6‐well plates to get 50% confluency after 12 h. Cells were transfected by identical plasmids for overall 48 h. And actinomycin D was added to 5 µg mL^−1^ at 6, 3, and 0 h before cell scraping collection. Total RNA was isolated by Trizol and analyzed by RT–PCR and RNA‐seq. For RNA‐seq, two rounds of mRNA selection were conducted and followed by TURBO DNase treatment. Then the mRNA was fragmented to about 100 nt. Sequencing libraries were prepared using NEBNext Ultra II RNA Library Prep Kit. mRNA lifetime profiling was generated from two biological replicates.

##### Dual‐Luciferase Reporter Assays

The dual‐luciferase reporter assay was performed as reported previously with some modification.^[^ ^38^ ^]^ Briefly, 100 ng wild‐type or mutant ASB2‐3′‐UTR, 50 ng GCN4‐dCas13b, 50 ng ScFv‐GGS^6^‐FTO (WT or Mut), 100 ng sgRNA and 20 ng pRL‐TK were co‐transfected into HEK‐293T cells in 24‐well plate. 48 h post transfection, the relative luciferase activities were accessed by Dual‐Luciferase Reporter Assay System (Promega, E1910). Each group was repeated in three biological replicates and two technical replicates.

##### Micro‐drop Digital PCR

HepG2.215 cells were seeded with 6‐well plate and the cell density was 1 × 10^6^ mL^−1^. Three plasmids (pFlagCMV2‐GCN4‐GGS^6^‐dCas13b, scFv‐GGS^6^‐FTO (WT or Mut) and pc0043‐HBV sgRNA) were co‐transfected into HepG2.215 cells at the ratio of 1:1:2, and the samples were collected 24 h later. The cellular DNA from each sample were treated with DNeasy Blood &Tissue Kit (QIAGEN GERMANY), then genomic DNA was removed by Plasmid‐Safe ATP‐Dependent DNase (PSAD, Epicentre USA) enzyme according to protocol. While small circular DNA could not be digested. Thus, cccDNA copy number was quantified using the QX200 Droplet Digital PCR system (Bio‐Rad, Hercules, CA) according to previous report.^[^ ^40^, ^41^ ^]^ Briefly, 20 µL ddPCR mixture consisted of 10 µL of 2 × ddPCR supermix for probes (Bio‐Rad), 950 nmol L^−1^ of cccDNA selective primers, 250 nmol L^−1^ of HBV cccDNA Taqman probe (5′‐FAMTCACCTCTGCCTAATCATCTCTAMRA‐3′), and 7 µL of DNA template. The mixture was loaded into the DG8 cartridge, 70 µL of droplet generation oil was added, and droplets (approximately 1 nL per droplet) were then formed in the droplet generator (Bio‐Rad). Generally, each sample could generate up to 20 000 stable water‐in‐oil droplets. Next, the droplets were transferred to a 96‐well PCR plate (Eppendorf) and amplified on a C1000 thermal cycler (Bio‐Rad) with a thermal profile beginning at 95 °C for 5 min, followed by 45 cycles of 94 °C for 30 s and 60 °C for 60 s, 1 cycle of 98 °C for 10 min, and ending at 4 °C. After amplification, the plate was loaded on the droplet reader (Bio‐Rad), and data were analyzed by QuantaSoft analysis software version 1.7.4 (Bio‐Rad) on the basis of Poisson distribution. For cell counting and normalization, *β*‐actin copy number in each DNA sample without PSAD treatment was also simultaneously determined with a pair of primers (5′‐ACTGTGCCCATCTACGAGG‐3′ and 5′‐CAGGCAGCTCGTAGCTCTT‐3′) and a probe (5′‐FAMCGGGAAATCGTGCGTGACTAMRA‐3′) in neighboring wells.

##### Co‐Immunoprecipitations

Cells were washed twice with cold PBS and then lysed with 500 mL cold Lysis Buffer (50 mm Tris‐HCl, pH 8.0, 150 mm NaCl, 2 mm EDTA, 1% NP‐40, 1 mm PMSF, protease inhibitors) for at least 5 min. Collect the lysates into EP tubes and incubate the lysates on ice for 30 min, pipetting every 5 min. After incubation, centrifuge the lysates at 15 000 × *g* for 10 min at 4 °C. Collect the supernatant and discard the pellet. Incubate 4 µg indicated antibody with 200 µL supernatant for 1 h at 4 °C. Immunoprecipitations were performed with anti‐His mouse antibody (ABclonal) or anti‐IgG mouse antibody (ABclonal). Then the supernatant‐antibody mix was bound to magnetic Dynabeads Protein A (Life Technologies) overnight at 4 °C, the immunoprecipitations were washed five times with Lysis buffer. The following antibodies were used for western‐blot analysis: anti‐Flag (rabbit, Sigma), anti‐His (mouse, ABclonal).

##### Data Analysis of NGS‐Data

All samples were sequenced by Illumina HiSeq XTen with paired end 150‐bp read length. The adapters were trimmed by using cutadapt^[^ ^42^ ^]^ (version 2.5) with parameters “‐m 18 –max‐n 3.” Reads that cannot be aligned to human rRNA sequences by using bowtie2^[^ ^43^ ^]^ with default parameters were mapped to human genome (GRCh38 and GENCODE.v29) by using STAR^[^ ^44^ ^]^ with parameters “–alignEndsType EndToEnd.” Uniquely mapped and proper‐paired alignments were filtered out by using bamtools with parameter “‐tag NH:1 ‐isProperPair true” and PCR amplification bias was eliminated by using picard (http://broadinstitute.github.io/picard; version 2.22.3) with parameters “MarkDuplicates REMOVE_DUPLICATES = true.” For m^6^A‐seq data, the fragment coverage of each base of all transcripts was calculated by custom script. The peak calling algorithm was modified from Ma et al.^[^ ^45^ ^]^ To calculate the enrichment score using the average fragment coverage of the window instead of the read count. The peak sequences in the length range of 50–200 nt were analyzed by using findMotifs.pl script of Homer^[^ ^46^ ^]^ (version 4.10) with parameters “‐rna ‐norevopp ‐mask ‐len 5,6,7” to find out the m^6^A motifs. For mRNA lifetime profiling data, we calculated the FPKM (Fragments Per Kilobase per Million) or all transcripts. The FPKM of 3 and 6 h were normalized to the FPKM of 0 h to calculate relative abundance and the relative abundance of 0 h were arbitrarily adjusted to 1. In order to meet the law of degradation, the relative abundance of 3 and 6 h were corrected as follows: 1) screeing out transcripts with length greater than 500‐nt, fragment count greater than 100 and FPKM greater 0.1 as representative transcripts; 2) sorting the relative abundance of 3 and 6 h of representative transcripts from small to large, respectively; 3) taking the 95th percentile value as normalization factor, respectively; 4) normalizing the relative abundance of 3 and 6 h to the corresponding factor. After these normalizations, it was ensured that the relative abundance of most genes at 3 and 6 h was lower than that at 0 h. we calculated the halftime of transcripts by linear fitting with the following formula (two replicates and three time points), where *T* represents the halftime, *A*_0_ represents the relative abundance of 0 h and always equal to 1, and *A_t_* represents the corrected relative abundance of 3 or 6 h:

(1) $$\begin{array}{c} A_{t}=A_{0}{\left(\frac{1}{2}\right)}^{\frac{t}{T}} \end{array}$$

(2) $$\begin{array}{c} \log_{2}\;A_{t}=-\frac{1}{T}\times t+\log_{2}\;A_{0} \end{array}$$

Let $k=-\frac{1}{T}$, *y* = log_2_ *A_t_*, and log_2_ *A*_0_ = 0, the simplified formula is shown as follows:

(3) $$\begin{array}{c} y=\mathrm{kt} \end{array}$$

(4) $$\begin{array}{c} T=-\frac{1}{k} \end{array}$$

##### GO Analysis

GO term analysis was performed by PANTHER tool, which was provided by Gene Ontology Consortium. Fisher's exact test was used to adjust the raw *p*‐value.

##### Statistical Analysis

All data are expressed as the mean ± standard error of the mean (SEM). For LC‐MS/MS, Dual‐Luciferase assay, qPCR quantification studies and western‐blot, *n* represents the number of independent experiments performed on different biological and/or technical replicates. Statistical analysis was performed using two‐tailed Student's *t*‐test (ttest_ind function of scipy package). Correlation analyses were performed using the Pearson correlation test. *p* < 0.05 was considered statistically significant. Symbol “*” represent *p* < 0.05, “**” represent *p* < 0.000, “***” represent *p* < 0.000001 and “NS” represent *p* > 0.05.

## Conflict of Interest

The authors declare no conflict of interest.

## Supporting information

Supporting Information
