# Supplementary material for: TRADES: Targeted RNA Demethylation by SunTag System
Source: Adv Sci (Weinh). 2020 Aug 16;7(19):2001402. doi: 10.1002/advs.202001402 (PMC7539198; doi:10.1002/advs.202001402)
Supplement: Supplementary file 1 — Supporting Information [file ADVS-7-2001402-s001.pdf]

© 2020 Wiley-VCH GmbH

ADVANCED SCIENCE Open Access

Supporting Information

for *Adv. Sci.,* DOI: 10.1002/advs.202001402

TRADES: Targeted RNA Demethylation by SunTag System

Jing Mo, Zonggui Chen, Shanshan Qin, Shu Li, Chuangang Liu, Lu Zhang, Ruoxi Ran, Ying Kong, Fang Wang, Songmei Liu, Yu Zhou, Xiaolian Zhang, Xiaocheng Weng, and Xiang Zhou**

© 2020 Wiley-VCH GmbH

Supporting Information

TRADES: Targeted RNA demethylation by SunTag system

Jing Mo, Zonggui Chen, Shanshan Qin, Shu Li, Chuangang Liu, Lu Zhang, Ruoxi Ran, Ying Kong, Fang Wang, Songmei Liu, Yu Zhou, Xiaolian Zhang, Xiaocheng Weng and Xiang Zhou**

J. Mo, S. Qin, Prof. X. Weng, Prof. X. Zhou
College of Chemistry and Molecular Sciences, Wuhan University, Wuhan 430072, China
E-mail: xcweng@whu.edu.cn; xzhou@whu.edu.cn
Z. Chen, Prof. Y. Zhou
The Institute for Advanced Studies, College of Life Sciences, State Key Laboratory of Virology, Wuhan University, Wuhan 430072, China
S. Li, C. Liu, L. Zhang, Y. Kong, Prof. X. Zhang
State Key Laboratory of Virology and Hubei Province Key Laboratory of Allergy and Immunology and Department of Immunology, School of Medicine, Wuhan University, Wuhan 430071, China.
R. Ran, Prof. S. Liu
Department of Clinical Laboratory, Center for Gene Diagnosis and Program of Clinical Laboratory, Zhongnan Hospital, Wuhan University, Wuhan, 430071, China.
Prof. F. Wang
Wuhan University School of Pharmaceutical Sciences, Wuhan, 430071, China.

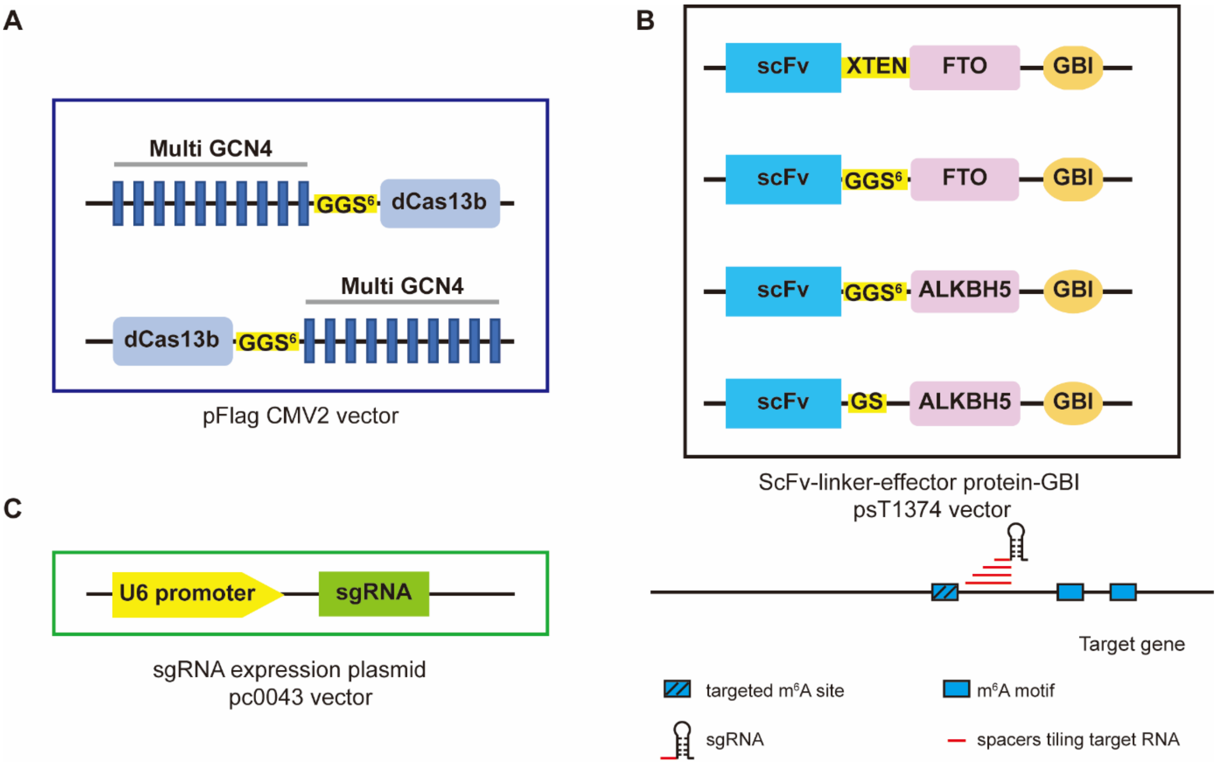

Figure S1 | The structure of plasmids used in this paper and the verification of their expression by Western-blots. (**A**) The schematic representation of plasmids. Multi-GCN4 peptides were inserted to the N-terminal or C-terminal of dCas13b. The vector used in this part is pFlag CMV2. (**B**) The schematic representation of plasmids used for demethylation. All these four kinds of plasmids have wild-type and mutant effector protein. The vector used in this part is psT1374. (**C**) sgRNA is the transcription product of U6 promoter in pc0043 vector. The red lines stand for the spacers tiling to the target RNA.

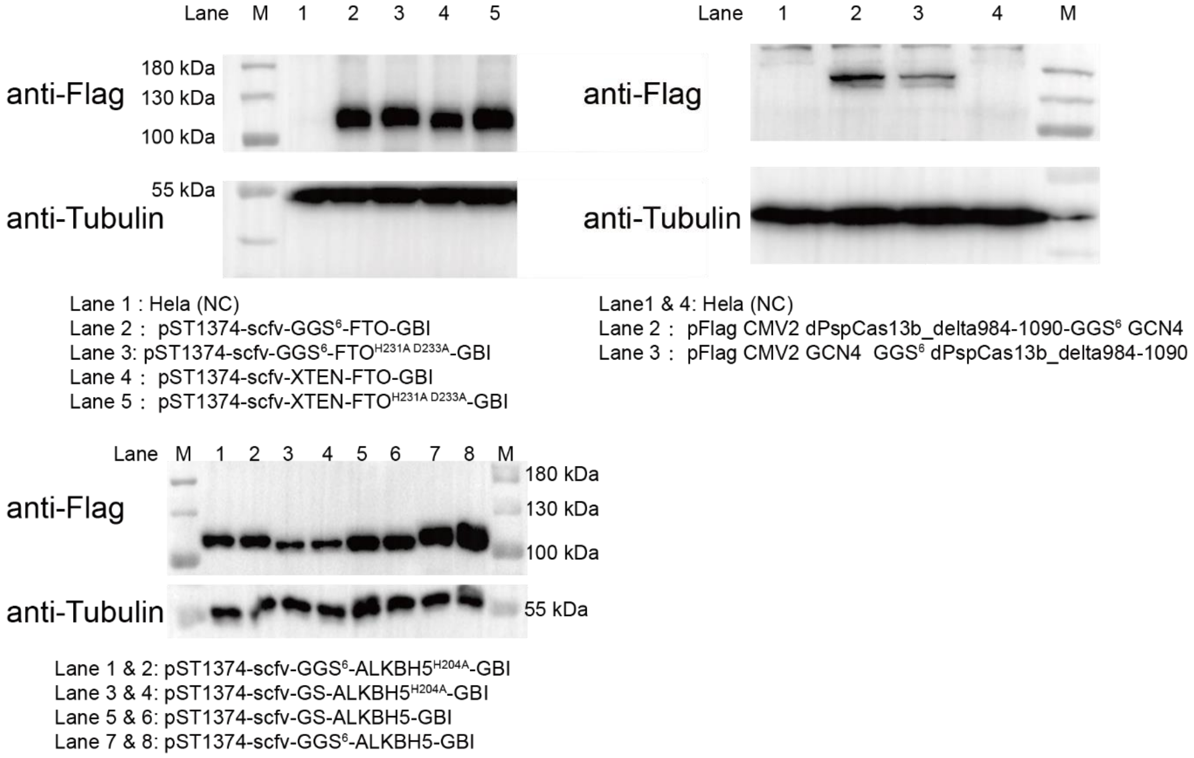

Figure S2 | The verification of recombinant protein expression by Western-blots. Protein expression of these recombinant plasmids transfected in HeLa cells. All plasmids were engineered with Flag tag. This experiment was repeated twice with similar results.

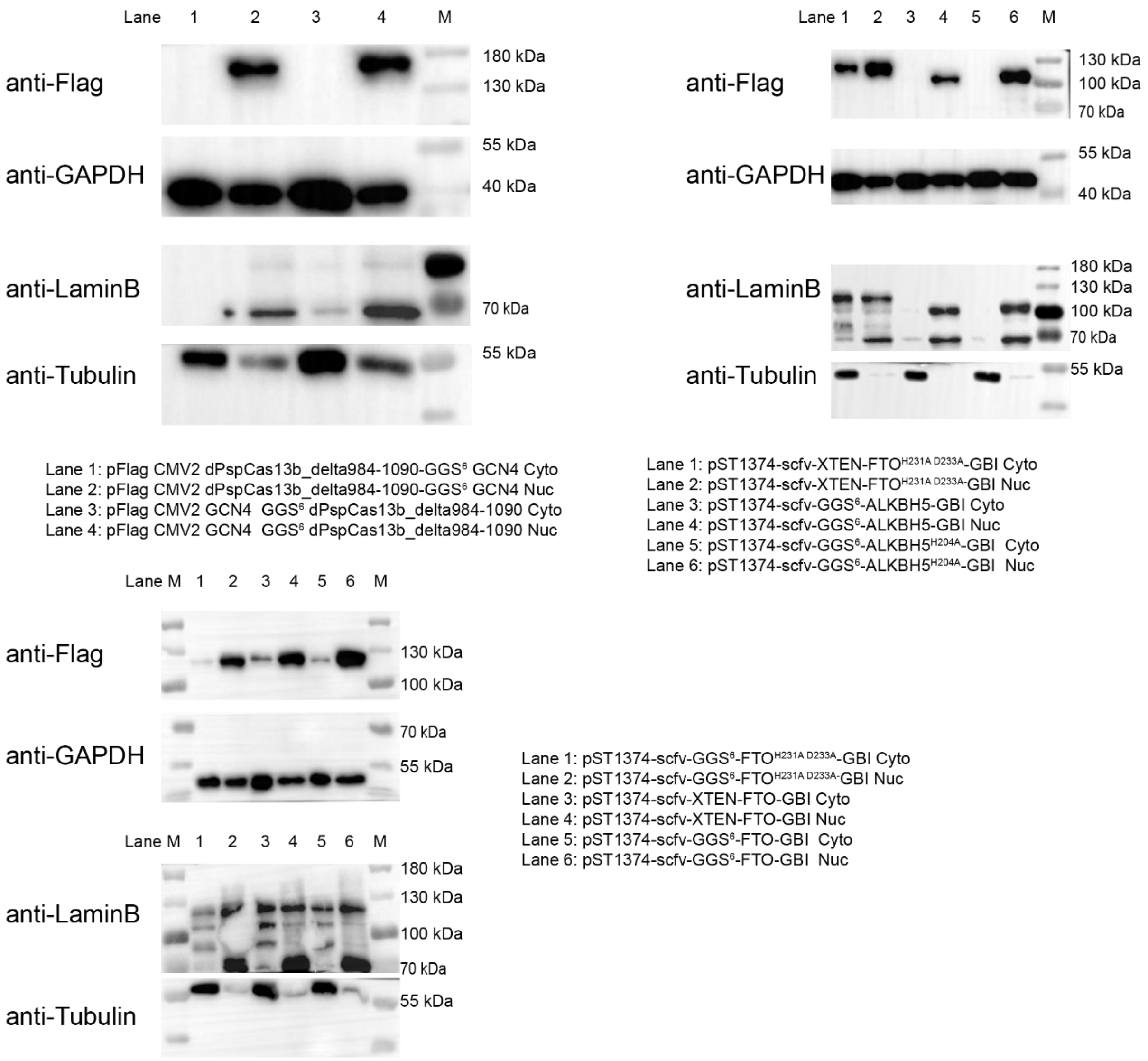

Figure S3 | Subcellular localization of these recombinant proteins in HeLa cells. Cyto means the cytoplasmic fraction of HeLa cells, Nuc means nuclear fraction of HeLa cells. The high expressions of these proteins in Nuc indicated that nuclear localization of these recombinant proteins. This experiment was repeated twice with similar results.

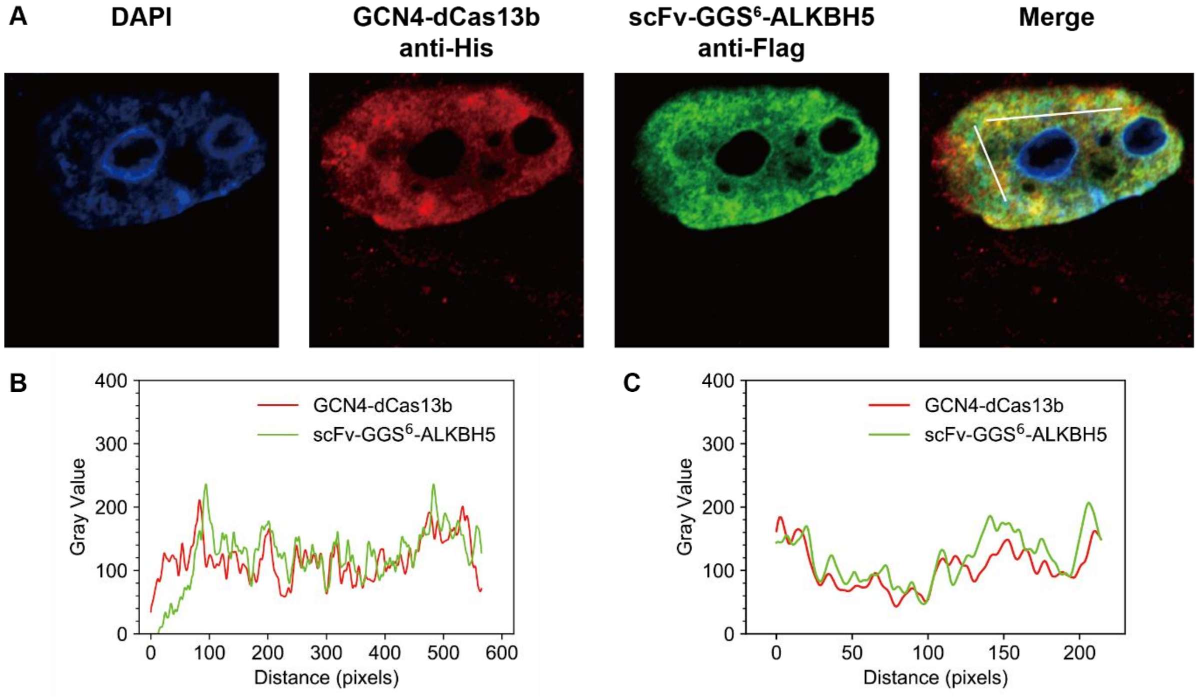

Figure S4 | The co-localization of dCas13b and RNA m⁶A demethylase by immunofluorescence staining. (**A**) GCN4-dCas13b, scFv-GGS^6-ALKBH5 were co-expressed in HeLa cells for overall 48 hours. Then cells were imaged using confocal microscopy. The GCN4-dCas13b plasmid was engineered with His-tag, while the scFv-GGS^6-ALKBH5 was engineered with Flag-tag. (**B**) Profile plots of gray value of GCN4-dCas13b (red) and scFv-GGS^6-ALKBH5 (green) corresponding to the region marked with white lines in Figure S4A (merge).

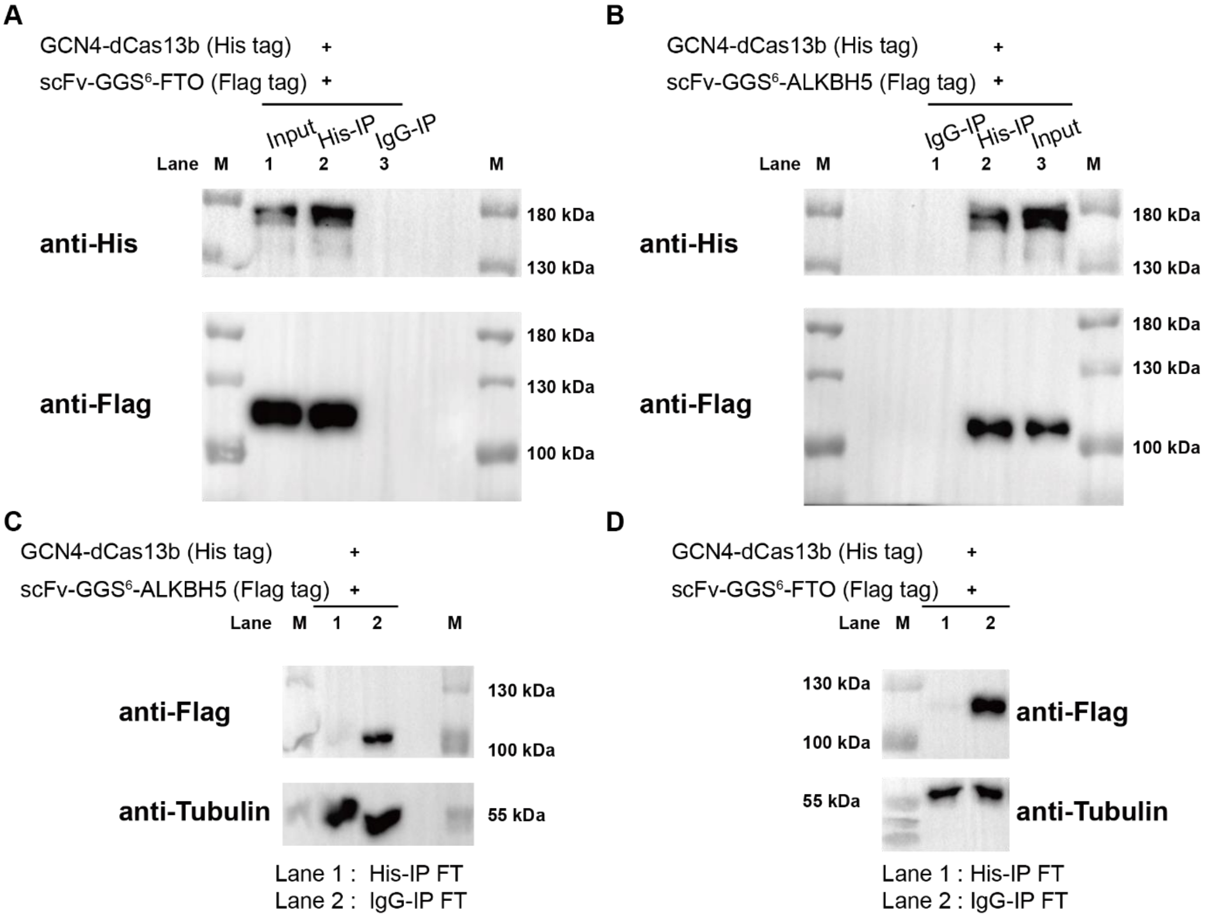

Figure S5 | GCN4-dCas13b could capture scFv-GGS^6-effector proteins in transfected cells. (**A**) Co-immunoprecipitation of the GCN4-dCas13b-interacting protein scFv-GGS^6-FTO. Western blot using the indicated antibodies with IgG used as control for the immunoprecipitation. (**B**) Co-immunoprecipitation of the GCN4-dCas13b-interacting protein scFv-GGS^6-ALKBH5. Western blot using the indicated antibodies with IgG used as control for the immunoprecipitation. (**C**) Flow through of Co-IP experiment shown in **A**. (**D**) Flow through of Co-IP experiment shown in **B**. These experiments were repeated four times with similar results.

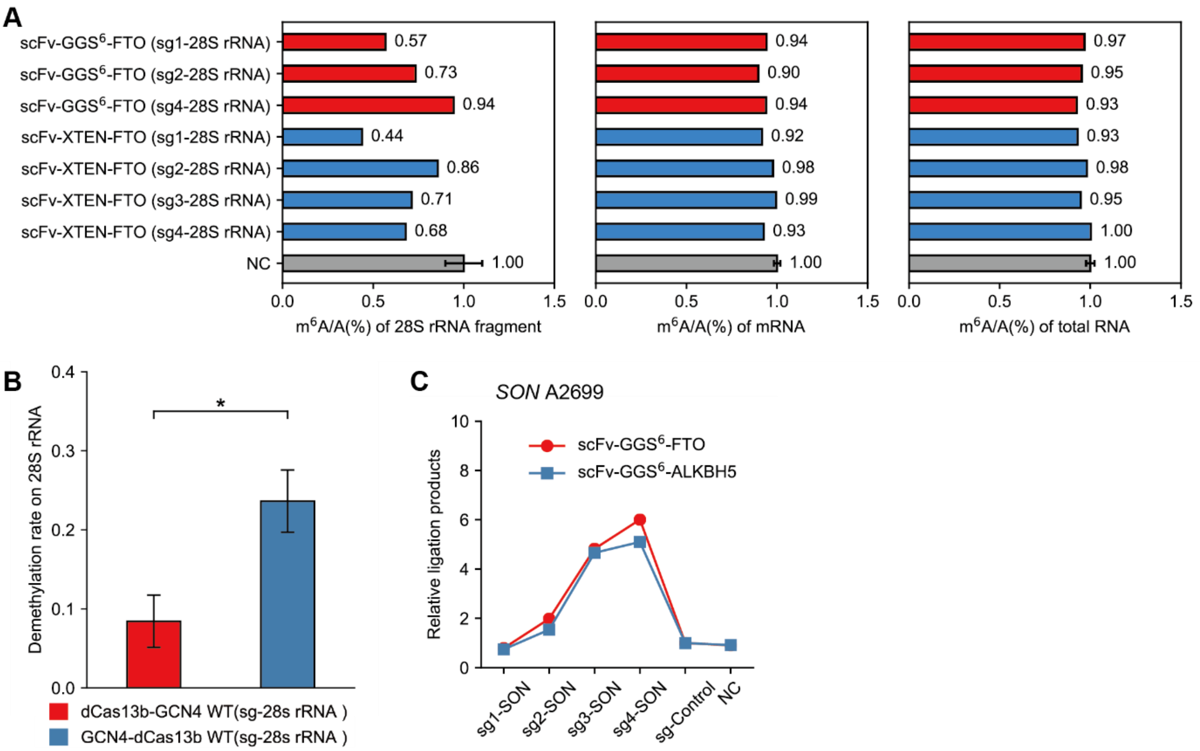

Figure S6 | Screen of sgRNA for 28S rRNA and SON. (**A**) m^6A/A (%) of 28S rRNA fragment, mRNA and total RNA in individual groups were quantified by LC-MS/MS. All groups excepted negative control (NC) were transfected with GCN4-dCas13b. ScFv-GGS^6-FTO or scFv-XTEN-FTO and sgRNA were transfected as indicated. For NC, bars represent mean ± SD from two independent experiments. (**B**) The demethylation rate on 28S rRNA fragment in individual groups were quantified by LC-MS/MS. All groups were transfected with scFv-XTEN-FTO and sg1-28S rRNA. GCN4-dCas13b or dCas13b-GCN4 was transfected as indicated. Bars represent mean ± SD from two independent experiments (n = 2, two-tailed Student's t-test). (**C**) Measurement of m^6A level on SON A2699 in individual groups. Relative ligation products of each group were quantified by qPCR. Cells excepted NC group were all transfected with GCN4-dCas13b. ScFv-effector proteins and sgRNA were transfected as indicated for overall 48 hours. NC stands for negative control.

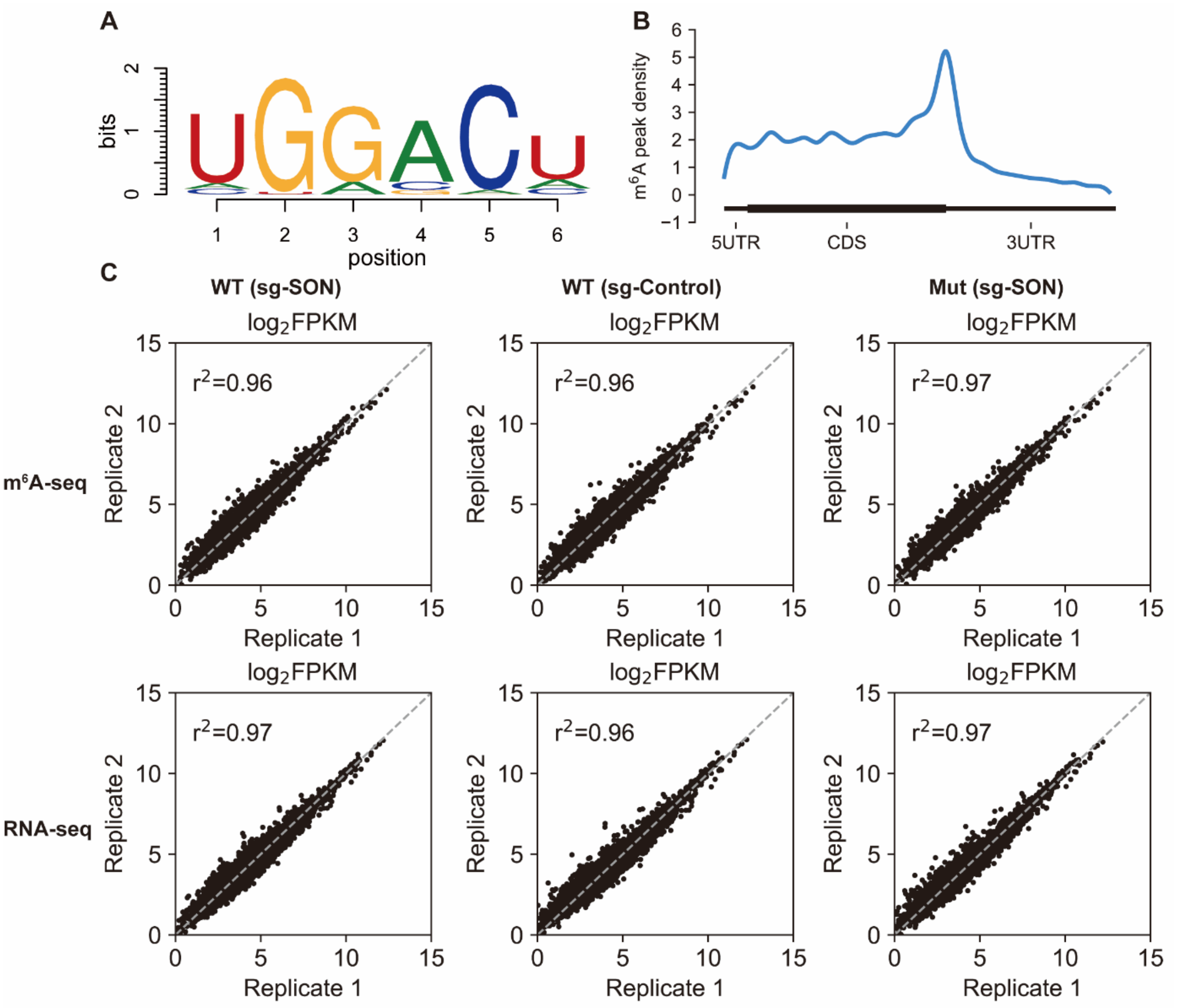

Figure S7 | The quality control of m^{6}A-seq and RNA-seq. (**A**) Binding motif identified by HOMER with m^{6}A-seq peaks. (**B**) Distribution of m^{6}A-seq peaks across the length of mRNA. Each region of 5'-UTR, CDS, and 3'-UTR were binned into different segments according to their average length. (**C**) Pearson correlation of RPKM between two biological replicates, in m^{6}A-seq or RNA-seq, respectively. In this part, cells were all transfected with GCN4-dCas13b. scFv-GGS^6-FTO (WT), scFv-GGS^6-FTO$^{H231A\ D233A}$ (Mut), sgRNA (sg-SON) or pc0043 vector (sg-Control) were transfected as indicated for overall 48 hours.

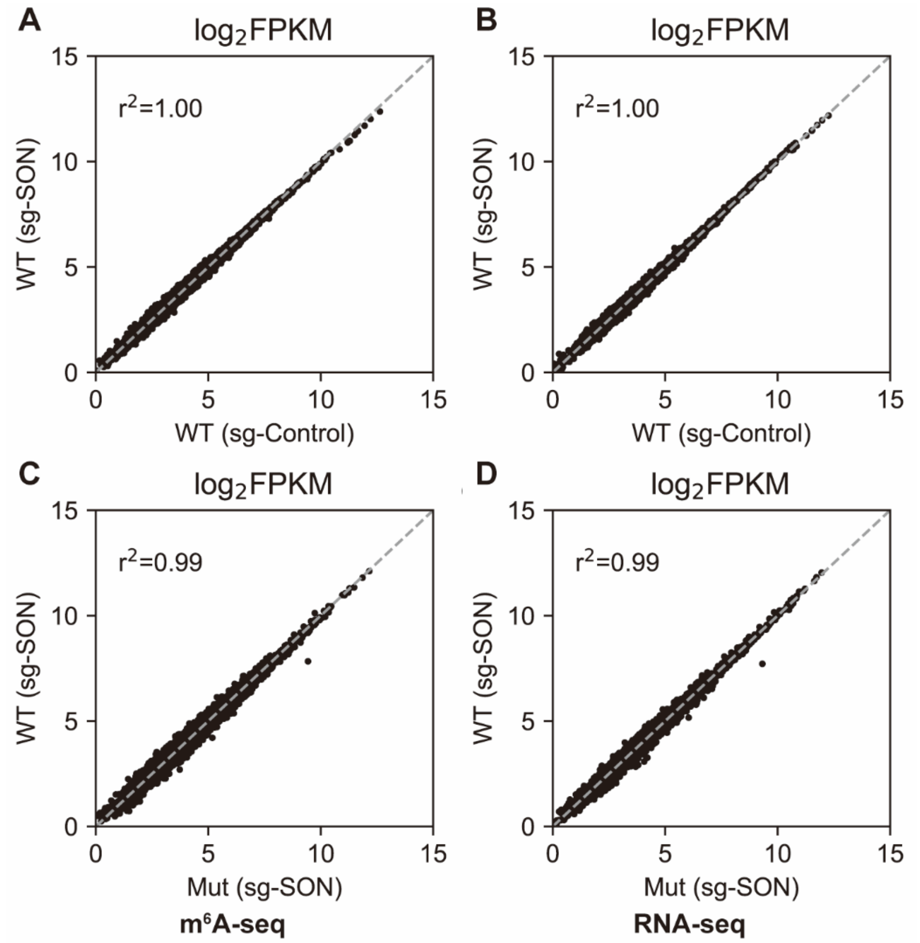

Figure S8 | TRADES performs good on-target capability. (**A**) Pearson correlation of RPKM between m^{6}A-enriched mRNA samples treated as indicate. (**B**) Pearson correlation of RPKM between input mRNA samples treated as indicate. (**C**) Pearson correlation of RPKM between m^{6}A-enriched mRNA samples treated as indicate. (**D**) Pearson correlation of RPKM between input mRNA samples treated as indicate.

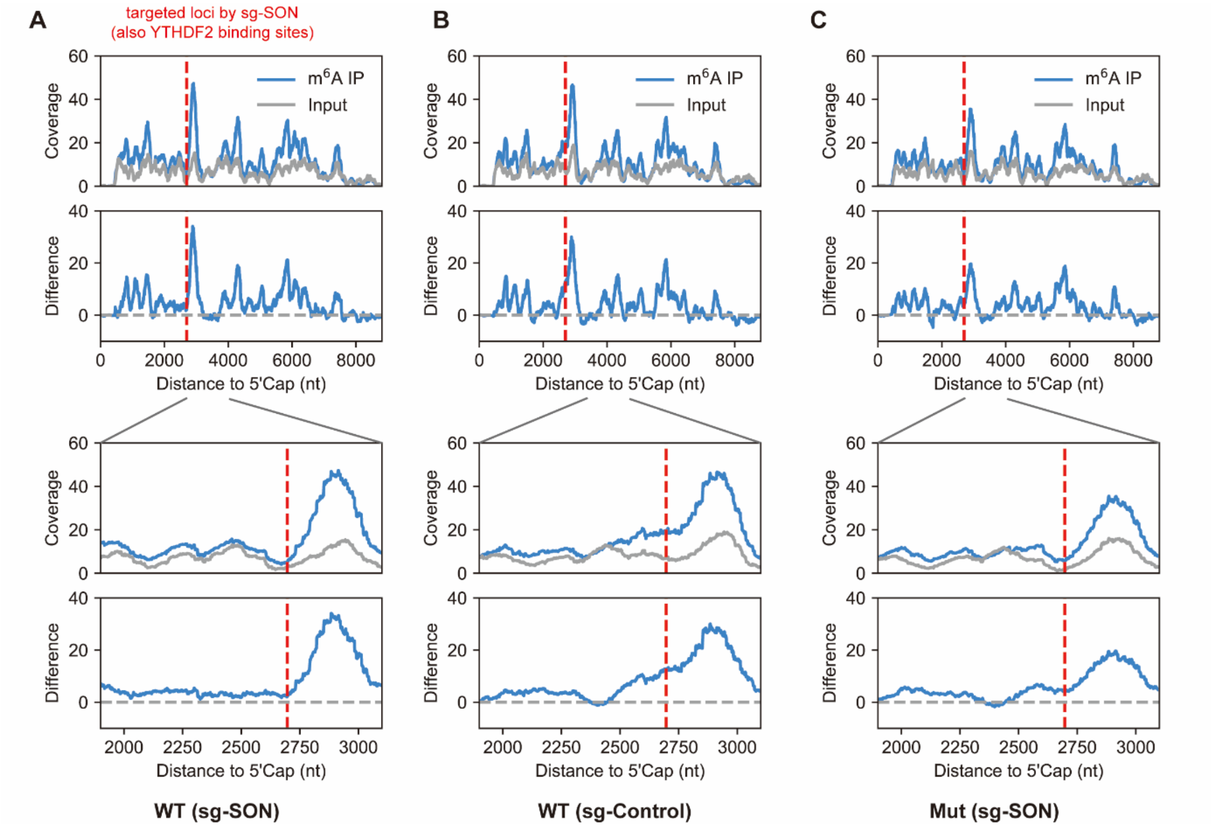

Figure S9 | TRADES performs good demethylation effect on SON A2699. (**A**) Comparison of normalized read coverages of SON mRNA between input mRNA and m^{6}A-enriced mRNA which are both from WT (sg-SON) treated samples. (**B**) Comparison of normalized read coverages of SON mRNA between input mRNA and m^{6}A-enriced mRNA which are both from WT (sg-Control) treated samples. (**C**) Comparison of normalized read coverages of SON mRNA between input mRNA and m6A-enriced mRNA which are both from Mut (sg-SON) treated samples. In this part, cells were all transfected with GCN4-dCas13b. scFv-GGS6-FTO (WT), scFv-GGS^6-FTO$^{H231A\ D233A}$ (Mut), sgRNA (sg-SON) or pc0043 vector (sg-Control) were transfected as indicated for overall 48 hours.

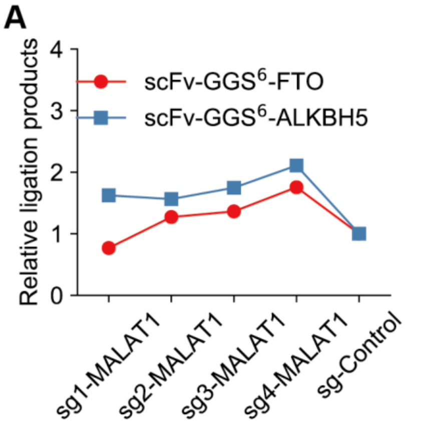

Figure S10 | Screen of sgRNA for MALAT1. (**A**) Measurement of m^6A level on MALAT1 A2577 in individual groups. Relative ligation products of each group were quantified by qPCR. Cells were all transfected with GCN4-dCas13b. ScFv-GGS^6-ALKBH5, scFv-GGS^6-FTO and sgRNA were transfected as indicated for overall 48 hours.

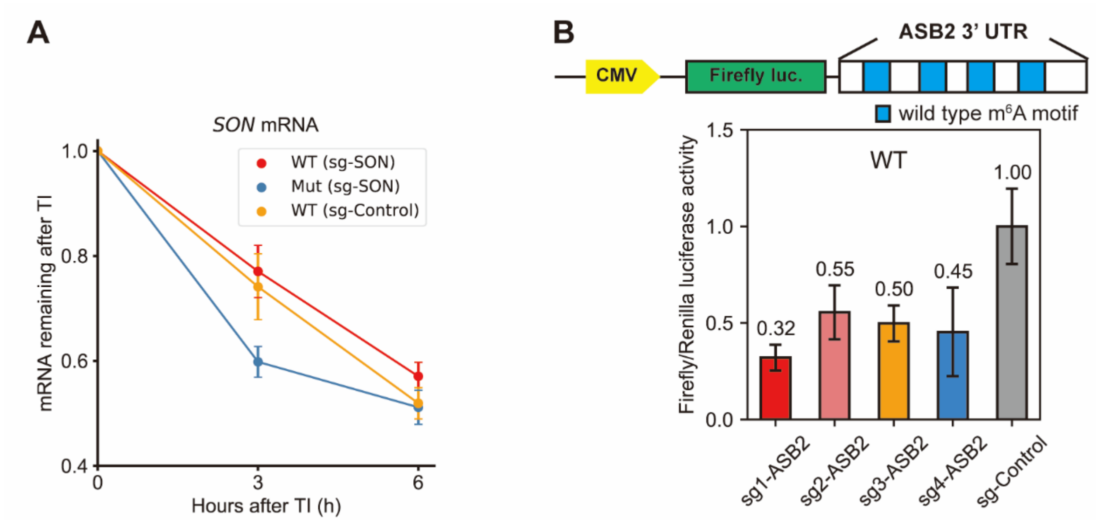

Figure S11 | Regulation of target RNA stability and expression in an m^{6}A-dependent manner using TRADES system. (**A**) The mRNA lifetime of SON in HeLa cells. Cells were all transfected with GCN4-dCas13b. scFv-GGS^6-FTO (WT), scFv-GGS^6-FTO$^{H231A\ D233A}$ (Mut), sgRNA (sg-SON) or pc0043 vector (sg-Control) were transfected as indicated for overall 48 hours. And actinomycin D was added to 5 μg/mL at 6 hours, 3 hours and 0 hour before cell scraping collection. The total RNA was isolated and then the SON abundance was analyzed by RT-qPCR. The mRNA remaining quantitative values at time 0 h were arbitrarily adjusted to 1. Scatters represent mean ± SD from two independent experiments (n = 2). (**B**) Schematic representation of constructs used in dual-luciferase analysis. ASB2 3'-UTR was cloned 3' downstream of the firefly luciferase. TRADES was applied to targeted demethylate ASB2 3'-UTR. The Firefly/Renilla luciferase activity was accessed by Dual-Luciferase Reporter Assay System. All groups were transfected with GCN4-dCas13b and scFv-GGS^6-FTO, the sgRNA was transfected as indicated. Bars represent mean ± SD from two independent experiments (n = 2).

Table S1: Primers used for subcloning.

Name	Sequence (5'->3')
XTEN-FTO-F1	5'-AGCGGCAGCGAGACTCCCGGGACCTCAGAGTCCGCCACACCCGAAAGTATGAAGCGCACCCCGACTGC-3'
XTEN-FTO-BamHI F2	5'-GTGGTGGTTCAGGAGGCGGCGGATCCAGCGGCAGCGAGACTCCCGG-3'
XTEN-FTO-RsrII R	5'-AGGATAAGCTTGTACTCTTCGGTCCGGGGTTTTGCTTCCAGAAGCT-3'
GGS^6-FTO-F1	5'-GGCGGTAGTGGAGGAAGCGGGGGGAGCGGAGGGAGTGGTGGATCCGGCGGCTCCATGAAGCGCACCCCGACTGC-3'
GGS^6-FTO-BamHI F2	5'-GTGGTGGTTCAGGAGGCGGCGGATCCGGCGGTAGTGGAGGAAGCGG-3'
GGS^6-FTO-RsrII R	The same as "XTEN FTO -RsrII R"
GGS^6-ALKBH5-F1	5'-GGCGGTAGTGGAGGAAGCGGGGGGAGCGGAGGGAGTGGTGGATCCGGCGGCTCCATGGCGGCCGCCAGCGGCTAC-3'
GGS^6-ALKBH5-BamHI-F2	5'-GTGGTGGTTCAGGAGGCGGCGGATCCGGCGGTAGTGGAGGAAGCGG-3'
GGS^6-ALKBH5-RsrII R	5'-CAGGATAAGCTTGTACTCTTCGGACCGGTGCCGCCGCATCTTCACCT-3'
$ALKBH5^{H204A}$-F	5'-CATCGTGTCTGCCGTGGACCCCATCCACATCTTCGAGCGCCCCATCGT-3'
$ALKBH5^{H204A}$-R	5'-TGGGGTCCACGGCAGACACGATGCAGCCGCCGGGCTGGTAGTCGTTGA-3'
C-GCN4-KpnI-F	5'-AATTGGTACCCCCAAGAAAAAACGCAAGGT-3'
C-GCN4-XbaI-R	5'-AATTTCTAGATCAATGGTGATGGTGATGAT-3'
N-dCas13b-HindIII-F	5'-AAGCTTATGCCTAAGAAAAAGAGGAAGGTGGAATTCATGAACATCCCCGCTCTGGT-3'
N-dCas13b-EcoRV-R	5'-AATTGATATCCTCTTTGGACACGA-3'
GGS^6-EcoRV-F	5'-ATCGGCGGTAGTGGAGGAAGCGGGGGGAGCGGAGGGAGTGGTGGATCCGGCGGCTCCGGTAC-3'

GGS^6-KpnI-R	5'-CGGAGCCGCCGGATCCACCACTCCCTCCGCTCCCCCCGCTTCCTCCACTACCGCCGAT-3'
N-GCN4-HindIII-F	5'-AATTAAGCTTCCCAAGAAAAAACGCAAGGT-3'
N-GCN4-EcoRV-F	5'-AATTGATATCACCGGTCACCTTGCGCTTCT-3'
C-dCas13b-KpnI-F	5'-AATTGGTACCCCTAAGAAAAAGAGGAAGGTGGAATTCATGAACATCCCCGCTCTGGT-3'
C-dCas13b-NotI-R	5'-AATTGCGGCCGCACATCCTCTTTGGACACGATGT-3'
Cut-His-FP	5'-TCGAAAGCCTATCCCTAACCCTCTTGA-3'
Cut-His-RP	5'-TCGATCAAGAGGGTTAGGGATAGGCTT-3'
His-tag-SacI-F	5'-CGTTTAGTGAACCGTCAGAATTGATCTACCATGCATCATCACCATCACCATA-3'
His-tag-HindIII-R	5'-AGCTTATGGTGATGGTGATGATGCATGGTAGATCAATTCTGACGGTTCACTAAACGAGCT-3'
28S sgRNA1 T	5'-CAACGGGACACCTGCGTTACCGTTTGACAGGTGTA-3'
28S sgRNA1 B	5'-CAACTACACCTGTCAAACGGTAACGCAGGTGTCCC-3'
28S sgRNA2 T	5'-CAACGGGACACCTGCGTTACCGTTTGACAGGTGTACCGCCCCAGTCAAACTCCCC-3'
28S sgRNA2 B	5'-CAACGGGGAGTTTGACTGGGGCGGTACACCTGTCAAACGGTAACGCAGGTGTCCC-3'
28S sgRNA3 T	5'-CACCGGGACACCTGCGTTACCGTTTGACAGGTGTACCGCCCCAGTCAAACTCCCCACCTGGCACTGTCCCCGGAG-3'
28S sgRNA3 B	5'-CAACCTCCGGGGACAGTGCCAGGTGGGGAGTTTGACTGGGGCGGTACACCTGTCAAACGGTAACGCAGGTGTCCC-3'
28S sgRNA4 T	5'-CACCGGGACACCTGCGTTACCGTTTGACAGGTGTACCGCCCCAGTCAAACTCCCCACCTGGCACTGTCCCCGGAGCGGGTCGCGCCCGG-3'

28S sgRNA4 B	5'-CACCCCGGGCGCGACCCGCTCCGGGGACAGTGCCAGGTGGGGAGTTTGACTGGGGCGGTACACCTGTCAAACGGTAACGCAGGTGTCCC-3'
MALAT1 sgRNA1 T	5'-CACCGTCAAAGTCCAATGCAAAAACATTAAGTTGG-3'
MALAT1 sgRNA1 B	5'-CAACCCAACTTAATGTTTTTGCATTGGACTTTGAC-3'
MALAT1 sgRNA2 T	5'-CACCGTCAAAGTCCAATGCAAAAACATTAAGTTGGTAATTACTCTTGATCTTGAA-3'
MALAT1 sgRNA2 B	5'-CAACTTCAAGATCAAGAGTAATTACCAACTTAATGTTTTTGCATTGGACTTTGAC-3'
MALAT1 sgRNA3 T	5'-CACCGTCAAAGTCCAATGCAAAAACATTAAGTTGGTAATTACTCTTGATCTTGAATTACTTCCGTTACGAAAGTC-3'
MALAT1 sgRNA3 B	5'-CAACGACTTTCGTAACGGAAGTAATTCAAGATCAAGAGTAATTACCAACTTAATGTTTTTGCATTGGACTTTGAC-3'
MALAT1 sgRNA4 T	5'-CACCGTCAAAGTCCAATGCAAAAACATTAAGTTGGTAATTACTCTTGATCTTGAATTACTTCCGTTACGAAAGTCCTTCACATTTTTCA-3'
MALAT1 sgRNA4 B	5'-CAACTGAAAAATGTGAAGGACTTTCGTAACGGAAGTAATTCAAGATCAAGAGTAATTACCAACTTAATGTTTTTGCATTGGACTTTGAC-3'
MALAT1 sgRNA-A T	5'-CACCGAATGCAAAAACATTAAGTTGGTAATTACTCTTGATCTTGAATTACTTCCGTTACGAAAGTCCTTCACATTTTTCAAACTAAGCT-3'
MALAT1 sgRNA-A B	5'-CAACAGCTTAGTTTGAAAAATGTGAAGGACTTTCGTAACGGAAGTAATTCAAGATCAAGAGTAATTACCAACTTAATGTTTTTGCATTC-3'
MALAT1 sgRNA-B T	5'-CACCGCATTAAGTTGGTAATTACTCTTGATCTTGAATTACTTCCGTTACGAAAGT-3'
MALAT1 sgRNA-B B	5'-CAACACTTTCGTAACGGAAGTAATTCAAGATCAAGAGTAATTACCAACTTAATGC-3'

MALAT1 sgRNA-C T	5'-CAACGAAGGCCTTAAATATAGTAGCTTAGTTTGAAAAATGTGAAGGACTTTCGTAACGGAAGTAATTCAAGATCAAGAGTAATTACCAC-3'
MALAT1 sgRNA-C B	5'-CACCGTCTTGATCTTGAATTACTTCCGTTACGAAAGTCCTTCACATTTTTCAAACTAAGCTACTATATTTAAGGCCTTCCAAATTCTTC-3'
SON sgRNA1 T	5'-CACCGCTGGGAGTCCATGGTGTTGGACGCTAGCAT-3'
SON sgRNA1 B	5'-CAACATGCTAGCGTCCAACACCATGGACTCCCAGC-3'
SON sgRNA2 T	5'-CACCGCTGGGAGTCCATGGTGTTGGACGCTAGCATTTGGGAGTCCATGGTGTTGG-3'
SON sgRNA2 B	5'-CAACCCAACACCATGGACTCCCAAATGCTAGCGTCCAACACCATGGACTCCCAGC-3'
SON sgRNA3 T	5'-CACCGCTGGGAGTCCATGGTGTTGGACGCTAGCATTTGGGAGTCCATGGTGTTGGATGCTAATATATGGGTCTCC-3'
SON sgRNA3 B	5'-CAACGGAGACCCATATATTAGCATCCAACACCATGGACTCCCAAATGCTAGCGTCCAACACCATGGACTCCCAGC-3'
SON sgRNA4 T	5'-CACCGCTGGGAGTCCATGGTGTTGGACGCTAGCATTTGGGAGTCCATGGTGTTGGATGCTAATATATGGGTCTCCATGGTGTTAGAAGC-3'
SON sgRNA4 B	5'-CAACGCTTCTAACACCATGGAGACCCATATATTAGCATCCAACACCATGGACTCCCAAATGCTAGCGTCCAACACCATGGACTCCCAGC-3'
ASB2 sgRNA1 T	5'-CACCGGCTACTCCTCTCTCCCCGTGGCCCCAGTTACTGGGTGTTCTCGTATTTCAGGTATCTAATCAGCCTGCCTGGGAGCGGCAAGGT-3'
ASB2 sgRNA1 B	5'-CAACACCTTGCCGCTCCCAGGCAGGCTGATTAGATACCTGAAATACGAGAACACCCAGTAACTGGGGCCACGGGGAGAGAGGAGTAGCC-3'

ASB2 sgRNA2 T	5'-CACCGGAGAGGGAGGCAGCCTGCAGCCTCGTCTGTCACCAGGTCCCCTTGGAGTTGGGAACACCGACGTCCTGAG-3'
ASB2 sgRNA2 B	5'-CAACCTCAGGACGTCGGTGTTCCCAACTCCAAGGGGACCTGGTGACAGACGAGGCTGCAGGCTGCCTCCCTCTCC-3'
ASB2 sgRNA3 T	5'-CACCGCAAATGATTCTTCTCCTTGACACATTCTGTTCTCTGCTCTGGCCAAAGCTCTGGGCCCTGAGACCCAGTG-3'
ASB2 sgRNA3 B	5'-CAACCACTGGGTCTCAGGGCCCAGAGCTTTGGCCAGAGCAGAGAACAGAATGTGTCAAGGAGAAGAATCATTTGC-3'
ASB2 sgRNA4 T	5'-CACCGGAGAAAGCTCTGAAGTCAAGTGGTGAGTTTTCCAGAACAAGTGGAGGGGCACAGGGAAGGCTCTGAGCACCACCTTCCCCAGAA-3'
ASB2 sgRNA4 B	5'-CAACTTCTGGGGAAGGTGGTGCTCAGAGCCTTCCCTGTGCCCCTCCACTTGTTCTGGAAAACTCACCACTTGACTTCAGAGCTTTCTCC-3'
HBV sgRNA1 T	5'-CACCGGTCAATGTCCATGCCCCAAAGCCACCCAAG-3'
HBV sgRNA1 B	5'-CAACCTTGGGTGGCTTTGGGGCATGGACATTGACC-3'
HBV sgRNA2 T	5'-CACCGGTCAATGTCCATGCCCCAAAGCCACCCAAGGCACAGCTTGGAGGCTTGAA-3'
HBV sgRNA2 B	5'-CAACTTCAAGCCTCCAAGCTGTGCCTTGGGTGGCTTTGGGGCATGGACATTGACC-3'
HBV sgRNA3 T	5'-CACCGGTCAATGTCCATGCCCCAAAGCCACCCAAGGCACAGCTTGGAGGCTTGAACAGTGGGACATGTACAAGAG-3'
HBV sgRNA3 B	5'-CAACCTCTTGTACATGTCCCACTGTTCAAGCCTCCAAGCTGTGCCTTGGGTGGCTTTGGGGCATGGACATTGACC-3'

HBV sgRNA4 T	5’-CACCGGTCAATGTCCATGCCCCAAAGCCACCCAAGGCACAGCTTGGAGGCTTGAACAGTGGGACATGTACAAGAGATGATTAGGCAGAG-3’
HBV sgRNA4 B	5’-CAACCTCTGCCTAATCATCTCTTGTACATGTCCCACTGTTCAAGCCTCCAAGCTGTGCCTTGGGTGGCTTTGGGGCATGGACATTGACC-3’
HIV sgRNA1 T	5’-CACCGATTGAGGCTGCGCCCATAGTGCTTCCTGCT-3’
HIV sgRNA1 B	5’-CAACAGCAGGAAGCACTATGGGCGCAGCCTCAATC-3’
HIV sgRNA2 T	5’-CACCGATTGAGGCTGCGCCCATAGTGCTTCCTGCTGCTCCCAAGAACCCAAGGAA-3’
HIV sgRNA2 B	5’-CAACTTCCTTGGGTTCTTGGGAGCAGCAGGAAGCACTATGGGCGCAGCCTCAATC-3’
HIV sgRNA3 T	5’-CACCGATTGAGGCTGCGCCCATAGTGCTTCCTGCTGCTCCCAAGAACCCAAGGAACAAAGCTCCTATTCCCACTG-3’
HIV sgRNA3 B	5’-CAACCAGTGGGAATAGGAGCTTTGTTCCTTGGGTTCTTGGGAGCAGCAGGAAGCACTATGGGCGCAGCCTCAATC-3’
HIV sgRNA4 T	5’-CACCGATTGAGGCTGCGCCCATAGTGCTTCCTGCTGCTCCCAAGAACCCAAGGAACAAAGCTCCTATTCCCACTGCTCTTTTTTCTCTC-3’
HIV sgRNA4 B	5’-CAACGAGAGAAAAAAGAGCAGTGGGAATAGGAGCTTTGTTCCTTGGGTTCTTGGGAGCAGCAGGAAGCACTATGGGCGCAGCCTCAATC-3’

Table S2: Primers used for RT-qPCR.

Name	Sequence (5’->3’)
qPCR-F for SELECT	5’-ATGCAGCGACTCAGCCTCTG-3’
qPCR-R for SELECT	5’-TAGCCAGTACCGTAGTGCGTG-3’
MALAT1 qPCR-F	5’-GACGGAGGTTGAGATGAAGCT-3’
MALAT1 qPCR-R	5’-ATTCGGGGCTCTGTAGTCCT-3’
SON qPCR-F	5’-GAGCCCCCAGTAGCAAAAGT-3’
SON qPCR-R	5’-CCGATGGTACGTCTACAGGC-3’
HPRT1 qPCR-F	5’-TGACACTGGCAAAACAATGCA-3’
HPRT1 qPCR-R	5’-GGTCCTTTTCACCAGCAAGCT-3’
GAPDH qPCR-F	5’-TGCACCACCAACTGCTTAGC-3’
GAPDH qPCR-R	5’-GGCATGGACTGTGGTCATGAG-3’
HBV qPCR-F	5’-GTCAACGACCGACCTTG-3’
HBV qPCR-R	5’-TGATTAGGCAGAGGTGAAAAAG-3’
ASB2 qPCR-F	5’-CGTGGTGCAGTTCTGTGAGT-3’
ASB2 qPCR-R	5’-GTGAGCCAGAGGTCTTGGAG-3’
HIV qPCR-F	5’-GGGCTAATTCACTCCCAACGA-3’
HIV qPCR-R	5’-ATCTGATCCCTGGCCCTGGT-3’

Table S3: Primers used in SELECT.

Name	Sequence (5'->3')
MALAT1 m^6A Up	5'-TAGCCAGTACCGTAGTGCGTGggatttaaaaaataatcttaactcaaag-3'
MALAT1 m^6A Down	5'-pos/ccaatgcaaaaacattaagtCAGAGGCTGAGTCGCTGCAT-3'
SON A2699 Up	5'-TAGCCAGTACCGTAGTGCGTGtggatgctagcatctgggag-3'
SON A2699 Down	5'-pos/ccatggtgttggacgctagcCAGAGGCTGAGTCGCTGCAT-3'
SON A2759 Up	5'-TAGCCAGTACCGTAGTGCGTGtagttgctaacatctgggag-3'
SON A2759 Down	5'-pos/ccatggtgctagacgctaacCAGAGGCTGAGTCGCTGCAT-3'
SON A2789 Up	5'-TAGCCAGTACCGTAGTGCGTGtagttgctaacatctgggag-3'
SON A2789 Down	5'-pos/ccatggagctagttgctaacCAGAGGCTGAGTCGCTGCAT-3'
SON A2819 Up	The same as "SON m^6A2789 Up"
SON A2819 Down	The same as "SON m^6A2789 Down"
HBV m^6A Up	5'-TAGCCAGTACCGTAGTGCGTGcaaattctttataagggtcaatg-3'
HBV m^6A Down	5'-pos/ccatgccccaaagccacccaCAGAGGCTGAGTCGCTGCAT-3'
ASB2-1 m^6A Up	5'-TAGCCAGTACCGTAGTGCGTGgtcctgagacttagtaagaagag-3'
ASB2-1 m^6A Down	5'-phos/ctgaggggctactcctctctCAGAGGCTGAGTCGCTGCAT-3'
HIV m^6A Up	5'-TAGCCAGTACCGTAGTGCGTGtaattgtctggcctgtaccg-3'
HIV m^6A Down	5'-phos/cagcgtcattgaggctgcgc-cagaggctgagtcgctgcat-3'
MALAT1 2515 m^6A Up	5'-TAGCCAGTACCGTAGTGCGTGaattacttccgttacgaaag-3'
MALAT1 2515 m^6A Down	5'-phos/ccttcacatttttcaaactaagCAGAGGCTGAGTCGCTGCAT-3'
MALAT1 2611 m^6A Up	5'-TAGCCAGTACCGTAGTGCGTGcagctgtcaattaatgctag-3'
MALAT1 2611 m^6A Down	5'-phos/cctcaggatttaaaaaataatcttaacCAGAGGCTGAGTCGCTGCAT-3'

Table S4: The exact sequences of the reporters used in dual-luciferase assay.
Wild type and mutant ASB2-3'-UTR were inserted into downstream of firefly luciferase of pMIR-REPORT vector (Luciferase miRNA Expression Reporter Vector, Ambion);
(1) ASB2-3’-UTR with wild-type m^6A sites:

ATGACTAGACTAGTGGCCACGGGGAGAGAGGAGTAGCCCCTC**AGACT**CTTCTTA

CTAAGTCTCAGGACGTCGGTGTTCCCAACTCCAAGGGGACCTGGTGACAGACGAG

GCTGCAGGCTGCCTCCCTCTCAGCCT**GGACA**GCTACCAGGATCTCACTGGGTCTC

AGGGCCCAGAGCTTTGGCCAGAGCAGAGAACAGAATGTGTCAAGGAGAAGAATC

ATTTGTTTAC**AAACT**GATGAGCAGATCCCAGACCTTCTCTACCTTCAGGAATGGC

AGAAACCTCTATTCCTGGGGCCAGGGCAGAGCTTGAGGTGTTCTGGGGAAGGTG

GTGCTCAGAGCCTTCCCTGTGCCCCTCCACTTGTTCTGGAAAACTCACCACTTGAC

TTCAGAGCTTTCTCTCCAA**AGACT**AAGATGAAGACGTGGCCCAAGGTAGGGGGT

AGGGGGAGCCTGGAAGCTTGGGATCTA

ASB2-3’-UTR with mutant m^6A sites:

ATGACTAGACTAGTGGCCACGGGGAGAGAGGAGTAGCCCCTC**AGTCT**CTTCTTA

CTAAGTCTCAGGACGTCGGTGTTCCCAACTCCAAGGGGACCTGGTGACAGACGAG

GCTGCAGGCTGCCTCCCTCTCAGCCT**GGTCA**GCTACCAGGATCTCACTGGGTCTC

AGGGCCCAGAGCTTTGGCCAGAGCAGAGAACAGAATGTGTCAAGGAGAAGAATC

ATTTGTTTAC**AATCT**GATGAGCAGATCCCAGACCTTCTCTACCTTCAGGAATGGC

AGAAACCTCTATTCCTGGGGCCAGGGCAGAGCTTGAGGTGTTCTGGGGAAGGTG

GTGCTCAGAGCCTTCCCTGTGCCCCTCCACTTGTTCTGGAAAACTCACCACTTGAC

TTCAGAGCTTTCTCTCCAA**AGTCT**AAGATGAAGACGTGGCCCAAGGTAGGGGGT

AGGGGGAGCCTGGAAGCTTGGGATCTA
